# Phenotypic characterization, plant growth and development, genome methylation, and mineral elements composition of neotetraploid lettuce (*Lactuca sativa* L.)

**DOI:** 10.3389/fpls.2023.1296660

**Published:** 2023-12-08

**Authors:** Ivan Simko, Rebecca Zhao

**Affiliations:** Sam Farr United States Crop Improvement and Protection Research Center, Agricultural Research Service, U.S. Department of Agriculture, Salinas, CA, United States

**Keywords:** lettuce, tetraploid, colchicine, development, DNA methylation, mineral element composition, pigments, tipburn

## Abstract

Stable neotetraploid lines of lettuce (*Lactuca sativa* L.) were produced from three phenotypically distinct cultivars (Annapolis, Eruption, Merlot) and an advanced breeding line (SM13-L2) using colchicine treatment of seeds or young seedlings. When tested under the greenhouse and field conditions, neotetraploids initially grew more rapidly than their diploid progenitors, however they reached their reproductive stage (bolting, flower bud formation, and flowering) substantially later. Seeds production on neotetraploids was delayed by more than 30 days compared to diploids. Tetraploid plants had fewer, but larger stomata and leaves, less chlorophyll per area, higher photosystem II photochemical efficiency, generally lighter root system, and produced less than 1% of seeds in comparison with diploids. Field-grown neotetraploids of all lines displayed a significant reduction in tipburn (1.8% vs. 22.2%, respectively), a highly undesirable physiological disorder. Changes in leaf and root mineral composition were detected in neotetraploids. Several elements were found in lower abundance than in diploids, most notably iron, calcium, and silicon. Whole genome bisulfite sequencing (WGBS) revealed 498 differentially methylated regions (DMR), with 106 of these regions having at least 50% difference in the level of methylation between neotetraploids and their diploid progenitors. At least 18 of the most prominent DMR were detected in proximity to genes predicted to be involved in plant development or reaction to biotic and abiotic stressors. Because neotetraploid lines have low seed production, they are not suitable for commercial cultivation. They can be used, however, in research to study the factors contributing to tipburn, traits affected by stomata size or density, and the effect of ploidy on resistance to environmental stressors.

## 1 Introduction

Natural polyploidy is generally considered to be involved in speciation with far-reaching evolutionary consequences (Van de Peer et al., 2021). Duplicated genes resulting from polyploidy appear to have been a key in domestication of agricultural crops and the evolution of their stress resistance (Renny-Byfield and Wendel, 2014). Resistance of polyploids to biotic and abiotic stressors may be a result of their increased genetic variation and the buffering effect of duplicated genes (Van de Peer et al., 2021). Ploidy manipulation and formation of induced polyploids has been incorporated into many plant-breeding programs since the creation of the first artificial polyploid (Winkler, 1916), though the degree of changes triggered by doubled chromosome number cannot be accurately predicted (Dermen and Emsweller, 1961). In mitotic polyploidization, the method routinely used by plant breeders, polyploidy can be artificially induced in somatic cells by doubling the number of their chromosomes (Manzoor et al., 2019). This type of chromosome duplication is frequently induced through application of anti-mitotic chemicals such as colchicine, the naturally occurring alkaloid. Colchicine applied to somatic cells interferes with microtubule formation, leading to creation of additional copies of chromosomes and existing genes. However, an application of colchicine may also lead to production of colchi-mutants, the plants with changed phenotypes due to cochicine-induced mutations (Datta, 2009).

Neopolyploids – an artificially produced polyploids (Comai, 2005) have to overcome difficulties in meiosis, genome restructuring, altered pattern of gene expression, and epigenetic reorganization (Comai, 2005; Soltis et al., 2015; Zhang et al., 2015) associated with changes in ploidy. In early generations, the progeny of neopolyploids show a high phenotypic variability due to occurrence of aneuploids, pseudoeuploids, and homeologue-recombinant genotypes (Ramsey and Schemske, 2002), and experience a frequent loss of duplicate gene copies (Soltis et al., 2015). Compared with their diploid progenitors, neotetraploids have generally slower growth, delayed and prolonged flower phenology, fewer but larger flowers, pollen grains, ovules, and seeds (Jaranowski and Kalasa, 1971; Ramsey and Schemske, 2002). Some polyploids also produce larger leaves, stems, and roots (Eng and Ho, 2019) due to increased cell size caused by the doubling of chromosomes, the phenomenon termed the “gigas” effect (Sattler et al., 2016).

Cultivated lettuce (*Lactuca sativa* L.) is an autogamous, diploid (*2n* = *2x* = 18) species from the *Asteraceae* (*Compositae*) family grown as vegetable in many areas around the world. Tetraploid genotypes of lettuce were previously developed through heat treatment (Einset, 1944; Einset, 1947), colchicine application (Thompson, 1939; Einset, 1944; Einset, 1947; Hiraoka, 1967; Eenink and Alvarez, 1975; Eenink, 1980; Reinink and Blom-Zandstra, 1989), and tissue culture regeneration from mesophyll protoplast (Engler and Grogan, 1984; Lelivelt et al., 2005). In comparison to their diploid progenitors, tetraploid plants had fewer but longer stomata, pollen grains, and seeds (Eenink and Alvarez, 1975; Eenink, 1980), larger flower heads (Eenink and Alvarez, 1975; Eenink, 1980), leaves (Thompson, 1939), and epidermal cells (Reinink and Blom-Zandstra, 1989), a lower concentration of nitrate and higher concentrations of organic acids and monosaccharides in plant sap (Reinink and Blom-Zandstra, 1989). Some authors reported, however, a lower fresh weight of tetraploid plants (Reinink and Blom-Zandstra, 1989), abnormal leaf and flower characteristics at maturity, smoother leaves that were lacking some of the savoying and marginal frilling characteristics for their progenitors, and delayed seed development (Thompson, 1939). A high frequency of cytological sterility occurring in lettuce tetraploids (Thompson, 1939; Engler and Grogan, 1984) was linked to undeveloped embryo sac or undeveloped ovule, or pollen not germinating or not growing on the styles (Einset, 1944).

We hypothesized that neotetraploid lines may have a more rapid initial development than their diploid counterparts, leading to earlier harvest of young plants intended for baby-leaf production (Simko et al., 2014b). Moreover, neotetraploid lines could contain different concentrations of compounds beneficial to human health, such as anthocyanins and chlorophyll. The objectives of the current study were to *de novo* generate tetraploid lines originating from several phenotypically distinct diploid cultivars and to compare performance of diploid and neotetraploid lettuce plants in more detail than was performed previously. Particularly, we evaluated plant growth in commercial fields, greenhouse conditions, and under different fertilization treatments. Detailed measurements were taken to compare seeds, leaves, stomata, content of pigments, performance of photosynthetic apparatus, roots, mineral elements composition, and the frequency of tipburn – an economically important physiological disorder. We have applied flow cytometry to determine plant ploidy and the whole genome bisulfite sequencing (WGBS) to identify differentially methylated regions (DMR) between diploid and tetraploid lines.

## 2 Materials and methods

### 2.1 Plant material

Three cultivars (Annapolis, Eruption, and Merlot) and one breeding line (SM13-L2) were used to generate lettuce neotetraploids. Cultivar Annapolis is a dark red romaine type, Eruption is red Latin, Merlot is red leaf, and SM13-L2 is a light green leaf type lettuce (Simko et al., 2014a). These accessions were included in the current study because of their frequent use in our breeding program owing to high resistance to one or more economically important diseases, including downy mildew (Simko et al., 2022), Impatiens Necrotic Spot Virus (Simko et al., 2023a), lettuce drop (Simko et al., 2023b), and Verticillium wilt race 1 (Inderbitzin et al., 2019).

### 2.2 Colchicine treatments and production of tetraploid lines

Seeds of each of the four accessions were split into two groups. One group of seeds was treated directly with colchicine (Sigma-Aldrich, St. Louis, MO, USA) solution, while seeds in the second group were germinated on wet filter paper (at 21°C) and three-days old seedling were treated with colchicine. Four combinations of colchicine concentration (0.2% and 0.3% w/v) and length of application (20 and 30 min.) were used for treating seeds. Seedlings were treated also with four, though nonidentical combinations of colchicine concentration (0.1% and 0.2% w/v) and length of application (10 and 20 min.). These combinations were based on results from our preliminary experiments. Either 200 seeds or 50 seedlings per accession were used in each of the treatment combinations.

Colchicine-treated seeds or seedlings were then laid on a filter paper to absorb the remaining colchicine solution. Seeds were subsequently planted into a plastic tray with a cell size of 2.5 × 2.5 cm. After 7-10 days, emerged seedlings were transplanted to 10.2 × 10.2 cm plastic pots. When freshy germinated seedlings, rather than seeds, were used for colchicine treatment, they were planted immediately after the treatment (and drying with filter paper) directly into 10.2 × 10.2 cm plastic pots. Plants from all treatments were cultivated in a growth chamber at 20°C and 16h light/8h dark photoperiod for a month. To produce seeds, plants were transplanted into 22.9-cm in diameter plastic pots and transferred to a greenhouse. The greenhouse temperature was set at 18-24°C, using only natural sunlight for illumination. Control, diploid plants from all accessions, were grown at the same conditions. Leaf samples were collected from about six-weeks old plants (both colchicine treated and diploid controls) and tested for ploidy via flow cytometry (Plantploidy.com, Columbus, OH, USA). Seeds produced on plants confirmed to be tetraploid were harvested. Harvested seeds were used to produce C1 to C4 generations of plants. In each generation, plants were retested for their ploidy using flow cytometry. Only confirmed tetraploid plants were used to produce seeds of the next generation. Seeds produced on C4 plants from stable, tetraploid lines were harvested and used in experiments. Due to seed, space, or cost limitations not all field and greenhouse experiments were performed with all four accessions. Reported results were obtained from at least three independent experiments (unless otherwise noted) and four to 25 plants per each experiment, ploidy, and accession combination.

### 2.3 Seedlings evaluations

Seeds of cultivars Annapolis and Eruption were placed on a wet filter paper and kept in a closed Petri dish. After four days at 21°C and 16h light/8h dark photoperiod, the whole germinated seedlings were weighted, their cotyledons removed and weighted separately. Epicotyl length, primary root length, and hypocotyl diameter were measured using digital caliper (World Precision Instrument 501601, Sarasota, FL, USA).

### 2.4 Greenhouse experiments

Plants were grown in plastic pots containing a 1:3 mix of sterilized Sungro Professional Growing Mix (Sungro Horticulture, Agawam, MA, USA) and sand, the greenhouse temperature was set at 18-24°C using the natural sunlight illumination. Plants were fertilized with granular slow-release fertilizer (Osmocote Plus 15-9-12, Scotts, Marysville, OH, USA) according to manufacturer’s instructions. Two pot sizes were used for experiments, either 15.2-cm in diameter containing approximately 1 kg of growing mix or 22.9-cm in diameter containing approximately 2.5 kg of growing mix. Smaller pots were usually used to grow plants harvested at earlier developmental stages, while larger pots were intended for plants grown into full market maturity or until seed production. Experiments focused on determining the effect of fertilizers on plant performance used two levels of Osmocote Plus 15-9-12 recommended by the manufacturer, low (40% of the optional amount) and high (160% of the optimal amount). All experiments were established using the completely randomized design with at least three replications. Plants were evaluated for the rate of their development, size (both above ground and roots), morphological characteristics, performance of photosynthetic apparatus, content of pigments, and mineral elements composition. More detailed descriptions of individual evaluations are provided below.

#### 2.4.1 Plant canopy

Canopy size was measured on plants of cultivars Annapolis and Eruption grown at the two levels (low and high) of fertilizer treatment. Overhead photographs of individual plants were taken with Nikon D5600 DSLR digital camera (Nikon Corporation, Tokyo, Japan) and analyzed using ImageJ2 v1.51 (Rueden et al., 2017). An absolute canopy area in cm^2^ was determined through the comparison with the standard of the known size.

#### 2.4.2 Plant development

The number of days after planting (DAP) were counted for a plant to reach the stage of bolting, flower buds forming, flowering, seed setting, and production of mature seeds. Plant development evaluations were assessed on cultivars Annapolis and Eruption grown in large pots.

#### 2.4.3 Number of leaves and leaf size

Number of leaves on a plant was determined on cultivars Annapolis, Eruption, Merlot, and breeding line SM13-L2 by counting all leaves longer than 3 cm. Leaf length was measured as a straight distance from the leaf base to its tip. Leaf width was determined by measuring the widest part of the leaf perpendicular to the leaf length. Leaf area was estimated with ImageJ v1.51 from a digital photograph of a flattened leaf. Leaf thickness was measured with calipers about 1 cm from the leaf tip avoiding major veins. The largest leaf on a plant was weighted immediately after removal from the plant to determine its fresh weigh. The leaf was then oven dried overnight at 70°C and weighted again to determine its dry weight.

#### 2.4.4 Content of pigments

Contents of chlorophyll and anthocyanins were assessed on plants of cultivars Annapolis, Eruption, Merlot, and breeding line SM13-L using hand-held meters SPAD-502 (Konica Minolta Sensing, Tokyo, Japan) and ACM-2000 plus (Opti-Sciences, Hudson, NH, USA), respectively. Three measurements per leaf were performed for each pigment approximately 2 cm from a leaf tip, avoiding major veins. Values were averaged and transformed according to previous recommendations (Simko, 2020), the square root transformation of SPAD values for chlorophyll (SPAD-Sqrt) and the binary logarithm transformation of ACI values for anthocyanins (ACI-Lb). Transformed data were used for statistical analyses.

#### 2.4.5 Photosynthetic apparatus

Performance of the photosynthetic apparatus was evaluated using maximum quantum yield (QY_Max), efficiency of photosystem II (Fv/Fm_L1), and normalized difference vegetation index (NDVI) (Myneni et al., 1995) parameters as previously described (Adhikari et al., 2019). Measurements were taken on three weeks old plants of cultivars Annapolis, Eruption, Merlot, and breeding line SM13-L using PlantScreen Transect XZ system and evaluated with FluorCam7 software (both from Photon Systems Instruments, Drasov, Czech Republic).

#### 2.4.6 Quantity and average weight of produced seeds

Seeds were harvested from mature plants of all four accessions grown in large pots. When a plant produced a large quantity of seeds, their number was estimated from the total weight of all seeds and the weight of 1,000 randomly selected seeds. If a plant produced less than 1,000 seeds, all seeds were counted, weighted, and the estimated weight of 1,000 seeds was calculated from these data.

#### 2.4.7 Number and size of stomata

Imprint of an adaxial leaf surface was taken using the common nail polish method (Forrest, 1962) that involves applying a thin layer of a clear nail polish on the leaf surface, drying the nail polish for about 5 min., applying a clear adhesive tape to the dry nail polish, removing the tape with nail polish imprint from the leaf, and mounting the imprint on a microscope slide for analysis. Counting of stomata and measuring of their length, width, and area was performed using Olympus BX60 microscope (Olympus Optical, Tokyo, Japan) and JENOPTIK GRYPHAX software v. 2.2.0.1234 (JENOPTIK Optical Systems, Jena, Germany). Stomata density (number of stomata per mm^2^) was calculated from the number of stomata per evaluated leaf area. Stomata were evaluated on both young and old leaves collected from the same plants of cultivars Annapolis, Eruption, Merlot, and breeding line SM13-L. Young leaves were those located close to the plant apex (longer than 5 cm) and old ones were those located close to the stem base.

#### 2.4.8 Plant size and biomass production

The aboveground size of a plant was assessed by measuring plant height (from plant base to apex) and width (maximum diameter of the plant). Biomass production was determined by plant fresh weight (immediately after harvest) and dry weight (after oven drying at 70°C for 24h). Plant size and biomass production was assessed on cultivars Annapolis and Eruption grown at the two levels (low and high) of fertilizer treatment.

#### 2.4.9 Roots size and biomass

Roots, together with potting mix, were carefully removed from pots, washed with tap water, and dried on a filter paper. The length of the longest root was measured and the whole root system was weighted to determine its fresh weight. Root system was then oven dried at 70°C overnight to determine its dry weight. Root size and biomass production was assessed on plants of cultivars Annapolis and Eruption grown at the two levels (low and high) of fertilizer treatment.

### 2.5 Field experiments

Plants of cultivars Annapolis and Eruption, and breeding line SM13-L2 were grown at the USDA-ARS research station in Salinas, CA, using the established agronomic practices for the production area (Simko et al., 2014b). Field experiments were set up in the randomized complete block design with three replications. Each plot contained between 25 to 30 plants of the same accession grown at the distance of ~30 cm between plants in a seedline. Plants were evaluated for the rate of their development using a 1–7 scale (1 = rosette; 2 = bolting-visible internode elongation; 3 = visible buds; 4 = expanded inflorescence; 5 = flowering–opening of first flower; 6 = more than half of buds flowered; 7 = mature seeds) (Rosental et al., 2021). At the market maturity, plant fresh weight, height, width, and the content of chlorophyll (SPAD-Sqrt) and anthocyanins (ACI-Lb) were evaluated as described for the greenhouse-grown plants. Harvested lettuce heads were then cut in half longitudinally to measure core length and for visual evaluations of tipburn (Macias-González et al., 2019). Frequency of tipburn was expressed as percent of plants showing symptoms from the total number of evaluated plants per accession.

### 2.6 Mineral elements composition

Content of 31 minerals, including three primary macronutrients (N, P, K), three secondary macronutrients (S, Ca, Mg), and 13 micro-nutrients (Fe, Mo, B, Cu, Mn, Na, Zn, Ni, Cl-, Co, Si, V, Se) was determined in leaves and roots of greenhouse-grown plants (cultivars Annapolis and Eruption), and in leaves of field-grown plants (cultivars Annapolis, Eruption, and breeding line SM13-L2). Approximately 5 g of fresh tissue was harvested and oven dried at 70°C for about 24 h. Samples were then shipped to Wallace Laboratories (El Segundo, CA, USA) for the analyses of mineral elements following the standard analytical methods. All analyses were performed on three plants per accession or a neotetraploid line.

### 2.7 DNA methylation in seeds

To determine whether differences in DNA methylation between progenitors and neotetraploid lines exist already in dormant seeds, harvested C4 seeds of cultivars Annapolis, Eruption, and breeding line SM13-L2 together with their respective neotetraploid lines were stored in identical conditions of -30°C and darkness for six months. The genomic DNA was extracted from seeds with Qiagen DNeasy Plant Mini Kit (Qiagen, Redwood City, CA, USA) and sequenced using Illumina HiSeq platform (Illumina, San Diego, CA, USA) by Genewiz (currently Azenta Life Sciences, South San Francisco, CA, USA). Detailed description of WGBS and subsequent analytical steps to identify DMR were provided previously (Simko, 2023). Briefly, cleaned reads with consistently high quality scores (Q > 30) were aligned to the Lsat_Salinas_v7 reference genome (Reyes-Chin-Wo et al., 2017) using Bismark 0.23.1 with bowtie2 2.4.2 (Krueger and Andrews, 2011). To identify DMR between diploids and tetraploids, the genome was tiled with a windows size of 1kb and a step size of 1kb. The number of methylated and the number of unmethylated CpG sites at the given region was obtained for individual accessions and pooled across all accessions within each ploidy level. The logistic regression model implemented in methylKit 1.4.1 (Akalin et al., 2012) followed by the SLIM method (Wang et al., 2011) was applied to compare the fraction of methylated CpG sites between diploids and neotetraploids. DMR with the adjusted experiment-wise *p*-value ≤ 0.05 and an absolute difference in methylation level between two ploidy levels ≥ 25% were annotated with TSS information from Refseq (https://www.ncbi.nlm.nih.gov/refseq/) (O'Leary et al., 2016). Genes were classified to be associated with DMR when the gene body or its 2-kb flanking region overlapped with DMR (Tong et al., 2021). DMR with adjusted experiment-wise *p*-value < 0.00015 and difference in methylation levels ≥ 50% were assigned to the group of pivotal DMR (PDMR) and used for more detailed analyses of predicted function using InterPro classification of protein families (https://www.ebi.ac.uk/interpro/) (Paysan-Lafosse et al., 2023), the Plant Proteome Database (http://ppdb.tc.cornell.edu/) (Sun et al., 2009), and UniProt (https://www.uniprot.org/) (The_UniProt_Consortium, 2023).

### 2.8 Statistical analyses

Phenotypic and composition data were analyzed using either *t*-test or two- or three-way analysis of variance (ANOVA) followed by *post-hoc* Tukey honestly significant difference (HSD) test. Main effect factors were ploidy, accession, and in fertilization experiments also the fertilizer level.

## 3 Results

### 3.1 Colchicine treatment

**Figure 1:**
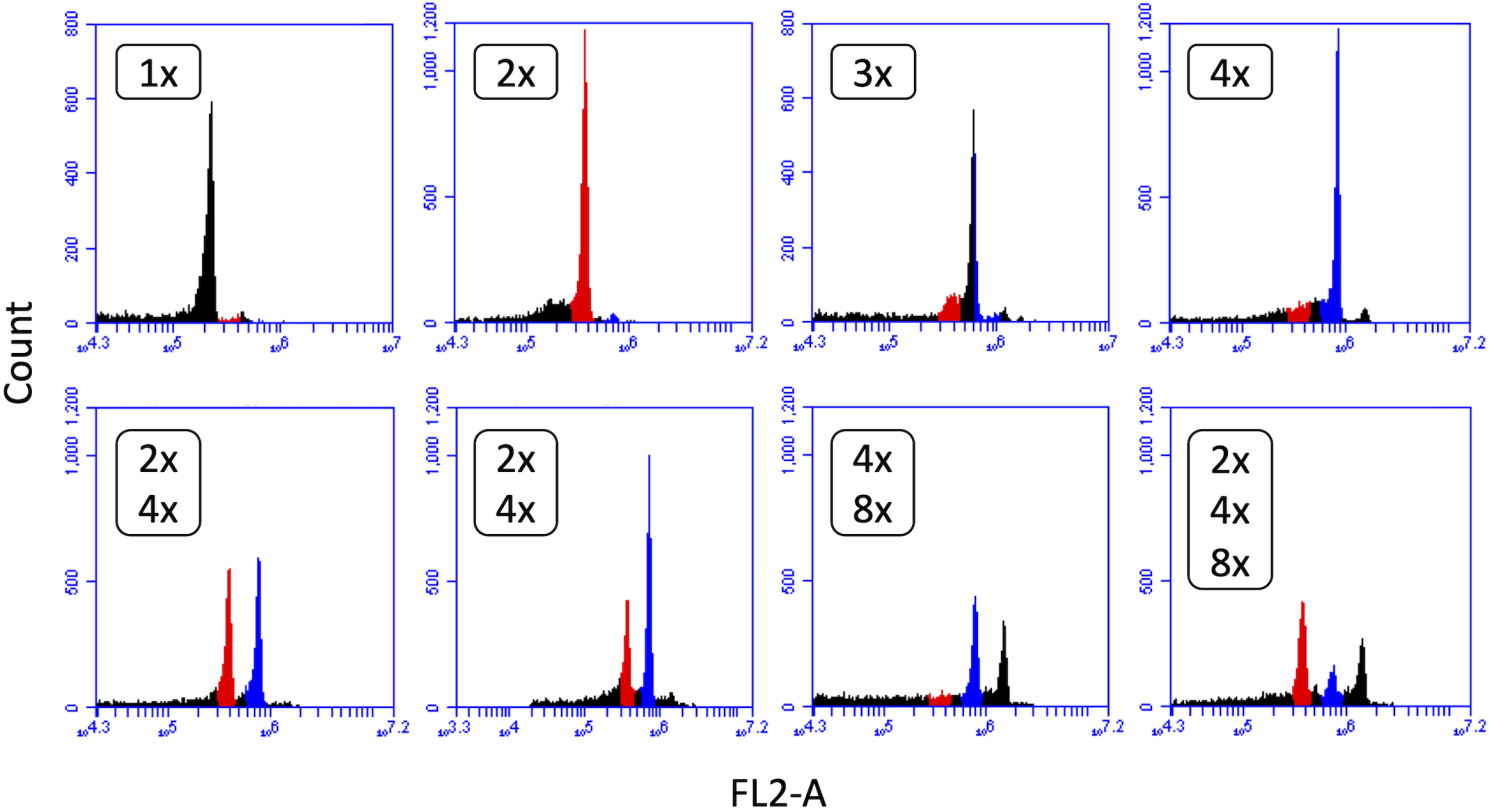
Flow cytometry analysis of lettuce ploidy. Detected ploidy levels and their combinations in lettuce plants treated with colchicine: 1x – monoploid, 2x – diploid, 3x – triploid, 4x – tetraploid, and 8x – octoploid. Only tetraploid (4x) lines were used for seed increase.

Colchicine-treated seeds that germinated were planted and ploidy of plants was determined approximately six weeks after planting with flow cytometry. Overall, 6.7% (Eruption) to 25.0% (SM13-L2) of plants showed tetraploid chromosome number. Other observed ploidy levels were monoploid (very rare), diploid, and triploid, with several plants being mixoploid (**Figure 1**). Only tetraploid plants were grown to full maturity and had their seeds harvested. Ploidy of plants in subsequent generations was assessed again with flow cytometry. Only plants that were confirmed to be stable tetraploids in all generations were selected for experiments. To minimize a chance that differences observed between diploids and tetraploids were caused by colchicine-induced mutations, experiments were performed only with neotetraploid lines that phenotypically resembled their respective diploid progenitors and produced highly uniform plants within the line.

### 3.2 Germination and size of seedlings

We did not notice any effect of ploidy on germination as all seeds germinated almost simultaneously. There was, however, a large difference in the initial size of seedlings four days after beginning of the germination test. Hypocotyl diameter, seedling weight, and cotyledon weigh were substantially larger (148% to 256% of diploid lines, respectively) in tetraploid lines (**Table 1**). In difference, epicotyl was similarly long on diploid (15.9 mm) and tetraploid (16.0 mm) plants, while primary roots on diploid plants were generally longer (25.8 mm vs. 21.7 mm).

**Table 1:** Size of seedlings after germination.

Trait	Annapolis			Eruption			Overall			4x/2x
	2x		4x	2x		4x	2x		4x	(%)
Epicotyl length (mm)	14.7	~	16.3	17.1	~	15.6	15.9	~	16.0	100.6
Primary root length (mm)	22.2	~	22.9	29.4	>	20.6	25.8	>	21.7	84.2
Hypocotyl diameter (mm)	0.63	<	0.90	0.59	<	0.91	0.61	<	0.91	148.3
Seedling weight (mg)	9.6	<	19.0	13.3	~	17.7	11.4	<	18.3	160.6
Cotyledon weight (mg)	2.7	<	6.0	2.0	<	6.0	2.3	<	6.0	256.1

Measurements were performed four days after placing seeds on a wet filter paper.

2x and 4x denote ploidy levels. Overall values combine data across tested accessions.

‘>‘ and ‘<‘ symbols indicate significant (p < 0.05) difference between two values and the direction of inequality, ‘~’ symbol indicates non-significant difference between the two values.

### 3.3 Greenhouse experiments

**Figure 2:**
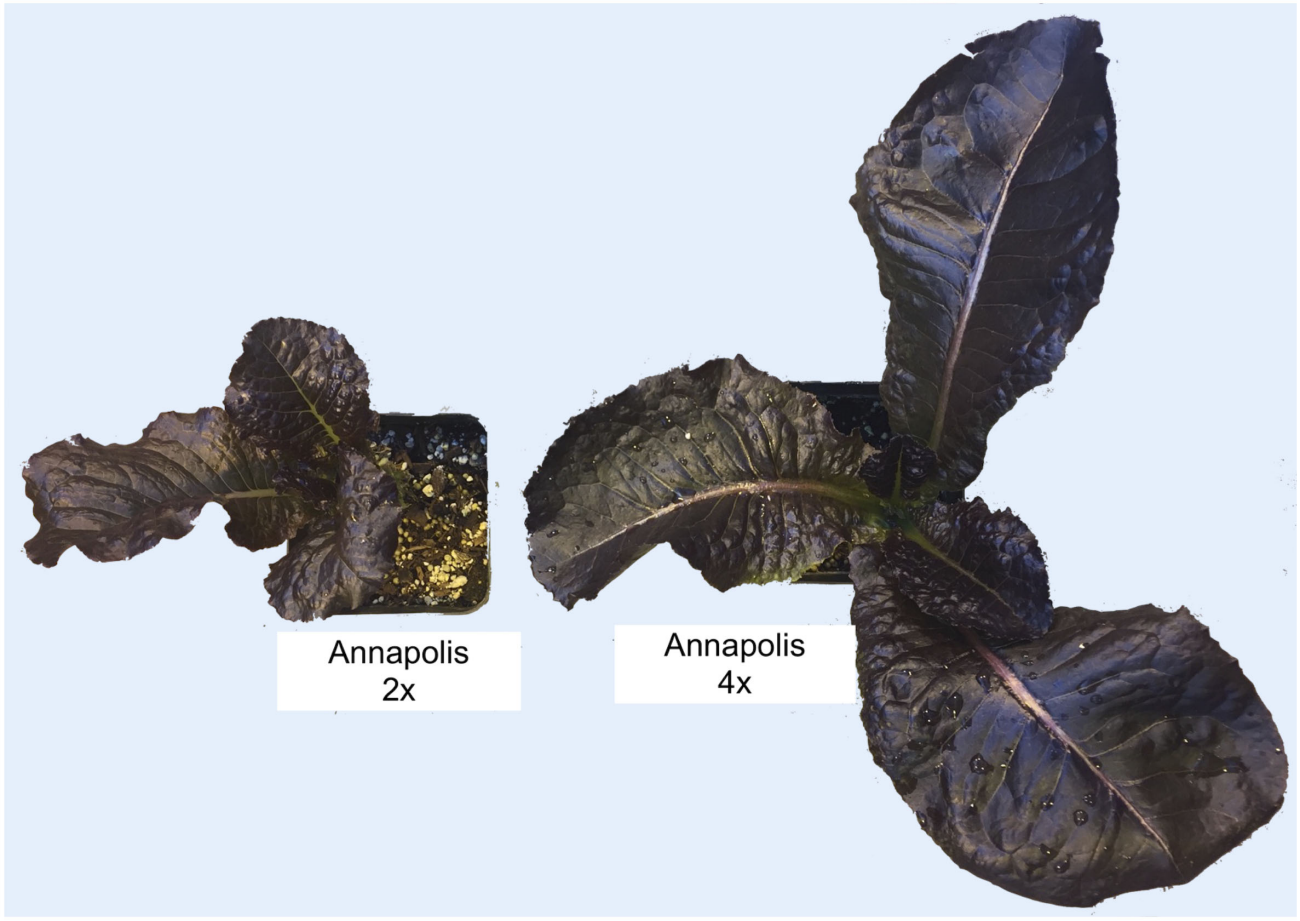
Approximately three weeks old diploid (2x) and neotetraploid (4x) plants of cultivar Annapolis. Tetraploid plant produced substantially larger leaves and grew more rapidly in early developmental stages. This particular tetraploid plant, however, did not yield any viable seeds for experiments. Please note that for a clear view the image background (concrete greenhouse floor) was removed, and labels were added with software, but no other alternation was made to the original photograph.

**Figure 3:**
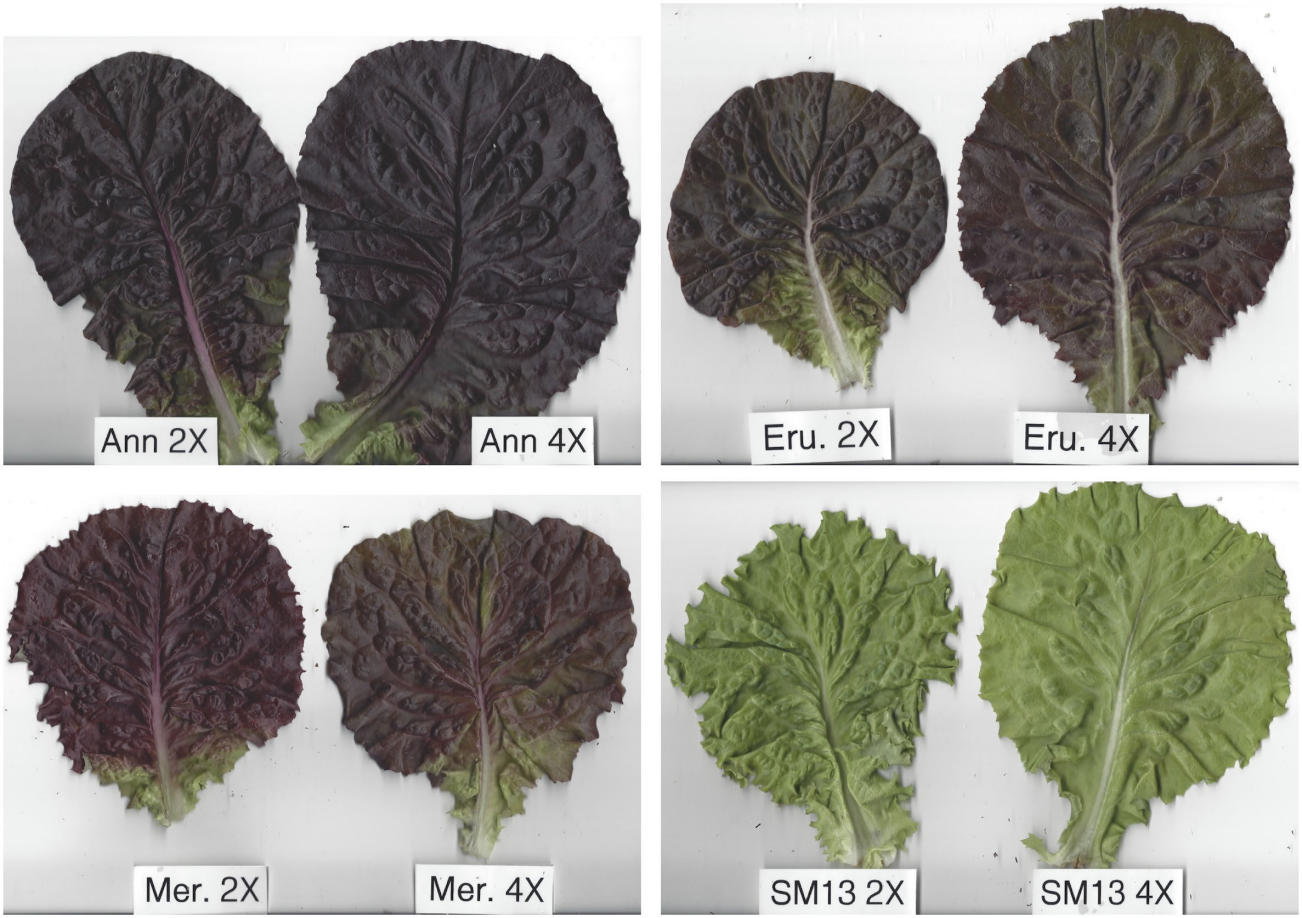
Comparison of largest leaves from four diploid (2x) accessions and their respective neotetraploid (4x) lines. Leaves collected from four weeks old, greenhouse-grown plants were placed under glass to obtain flat profiles and photographed. ‘Ann’ is cultivar Annapolis, ‘Eru’ is cultivar Eruption, ‘Mer’ is cultivar Merlot, and ‘SM13’ is breeding line SM13-L2.

**Figure 4:**
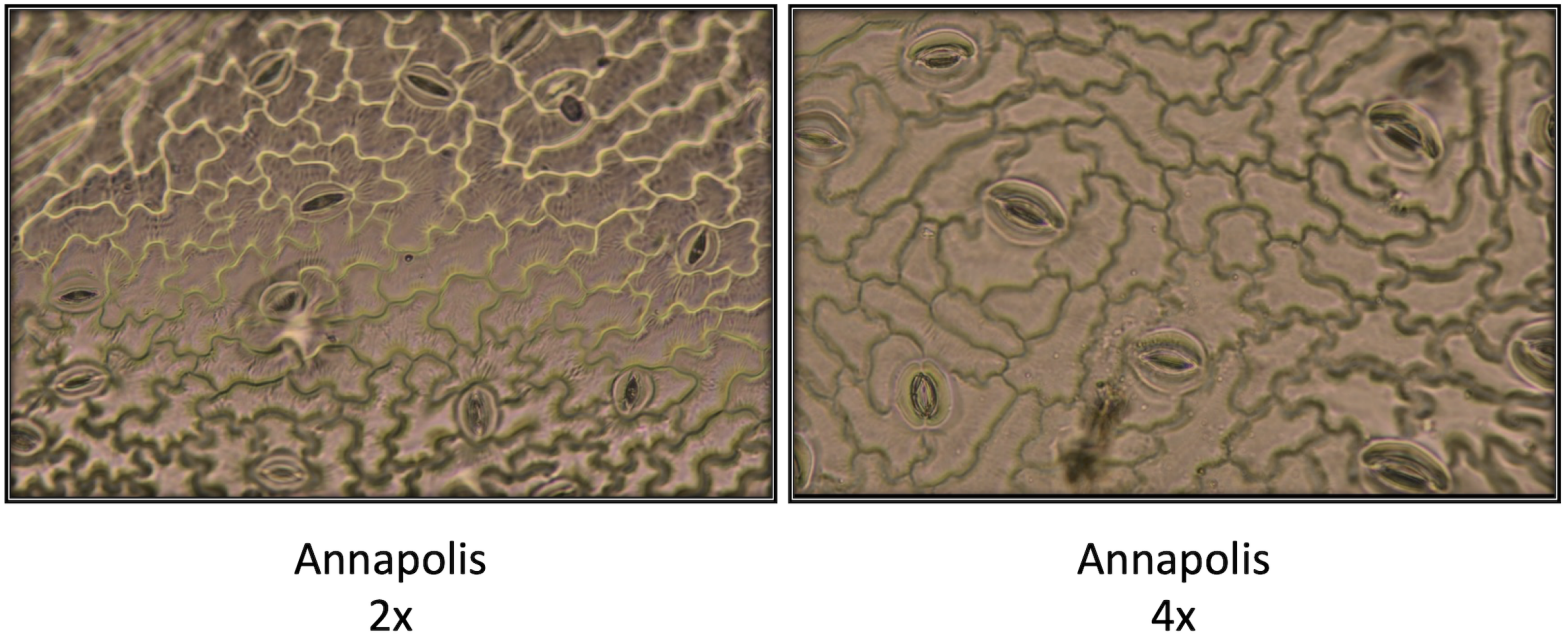
Comparison of size and density of stomata on diploid (2x) and neotetraploid (4x) leaves of cultivar Annapolis. Photographs were taken from imprints of adaxial leaf surfaces. Imprints were made on young leaves of four weeks old plants grown in a greenhouse.

In early stages, tetraploid plants cultivated in a greenhouse grew faster than diploid plants (**Figure 2**; **Table 2**), with their canopies being significantly larger at 28 DAP (132%), 32 DAP (129%), and 39 DAP (119%) after planting. Later (45 DAP and 56 DAP), the canopy areas of diploid and tetraploid plants were similarly large, though tetraploid Eruption line still had a significantly larger canopy than its diploid progenitor when fertilized at the high rate (**Table 2**). As plant growth progressed, the development of tetraploid plants slowed compared with their diploid counterparts (**Table 3**). Tetraploids reached significantly later the stages of bolting (10 days later), flower bud formation (13 days later), flowering (12 days later), seed setting (20 days later), and seed maturity (37 days). Because of a high sterility rate, tetraploid lines produced only about 0.6% of seeds compared with diploids (**Table 3**), though the weight of individual seeds was significantly larger (1.05 g per 1,000 seeds vs. 1.78 g, respectively) (**Table 4**). Flower heads of all tetraploid lines appeared to be visually larger than those of diploid accessions, however, no measurements were taken to obtain quantitative data for statistical analyses. Tetraploid plants also had a fewer (12.3 vs. 7.4, respectively) but significantly larger leaves (length 110.7%, width 109.1%, area 123.0%, and fresh weight 135.1%) (**Figure 3**; **Table 4**). No significant difference was detected in performance of the photosynthetic apparatus using maximum quantum yield (QY_Max) and normalized difference vegetation index (NDVI) parameters, though efficiency of photosystem II (Fv/Fm) was higher in tetraploid lines (**Table 4**). Tetraploid plants had, however, a lower concentration of both chlorophyll (SPAD-Sqrt of 5.4 vs. 5.1, respectively) and anthocyanins (ACI-Lb of 4.0 vs. 3.7, respectively), though only difference in the content of chlorophyll was significant (**Table 4**). When stomata on young and old leaves were compared, results were highly consistent (**Table 5**). On both young and old leaves, tetraploid plants had significantly fewer stomata per area than diploids (79.1% on young leaves and 57.7% on old leaves), but the size of an individual stoma was much larger (stoma area 165.7% on young leaves and 185.6% on old leaves) (**Figure 4**). The differences in leaf number, leaf size, and chlorophyll and anthocyanin content were confirmed when diploid and tetraploid plants grown at both low and high fertilizer treatment were compared (**Table 6**). In addition, it was observed that leaves of tetraploid plants were generally thicker (225 µm vs. 236 µm), and plants were slightly, but significantly taller (18.5 cm vs. 19.5 cm) and wider (27.7 cm and 29.2 cm), though their fresh and dry weights were similar as those of diploids (**Table 6**). The length of the main root was similar in diploid (21.8 cm) and tetraploid (22.7 cm) plants; however, the root system of tetraploid plants was significantly lighter both at fresh (87.1%) and dry weight (88.3%) (**Table 6**).

**Table 2:** Canopy area of greenhouse-grown plants cultivated at two fertilizer rates.

Days after planting	Annapolis			Annapolis			Eruption			Eruption			Overall			
(DAP)	Low rate			High rate			Low rate			High rate						4x/2x
	2x		4x	2x		4x	2x		4x	2x		4x	2x		4x	(%)
28	8.1	~	8.2	7.7	<	15.8	7.8	~	9.4	12.5	~	14.1	9.0	<	11.9	131.6
32	40.9	~	33.5	36.8	<	52.5	53.0	<	71.9	50.1	<	75.2	45.2	<	58.3	128.9
39	115	~	125	152	~	161	145	~	186	159	~	205	143	<	169	118.6
45	494	~	441	324	~	317	361	~	415	322	<	451	375	~	406	108.2
56	991	~	620	1,040	~	924	363	~	397	553	<	1,060	737	~	750	101.8

Canopy area in cm^2^.

Low fertilizer rate (40% of optimal), high fertilizer rate (160% of optimal).

2x and 4x denote ploidy levels. Overall values combine data across tested accessions.

‘>‘ and ‘<‘ symbols indicate significant (p < 0.05) difference between two values and the direction of inequality, ‘~’ symbol indicates non-significant difference between the two values.

**Table 3:** Development and seed production on greenhouse-grown plants.

Developmental stage or seeds quantity	Annapolis			Eruption			Overall			4x/2x
	2x		4x	2x		4x	2x		4x	(%)
Bolting (DAP)	57.1	<	63.8	60.1	<	74.3	58.6	<	69.0	117.7
Flower buds forming (DAP)	65.9	<	78.8	83.6	<	96.5	74.8	<	87.6	117.1
Flowering (DAP)	84.2	<	96.8	100.6	<	111.2	92.4	<	104.0	112.6
Seed setting (DAP)	100.0	<	119.0	111.4	<	132.3	105.7	<	125.7	118.9
Production of mature seeds (DAP)	113.5	<	145.3	127.8	<	169.6	120.7	<	157.5	130.5
Number of seeds	13,750.0	>	59.8	6,520.0	>	57.7	10,135.0	>	58.7	0.6

DAP – days after planting.

2x and 4x denote ploidy levels. Overall values combine data across tested accessions.

‘>‘ and ‘<‘ symbols indicate significant (p < 0.05) difference between two values and the direction of inequality, ‘~’ symbol indicates non-significant difference between the two values.

**Table 4:** Leaf size, pigments content, performance of the photosynthetic apparatus, and average seed weight on greenhouse-grown plants.

Trait	Annapolis			Eruption			Merlot			SM13-L2			Overall			4x/2x
	2x		4x	2x		4x	2x		4x	2x		4x	2x		4x	(%)
Leaf																
Number of leaves (> 3 cm)	10.3	>	7.6	13.1	>	9.4	12.9	>	6.8	13.1	>	7.0	12.3	>	7.4	60.2
Maximum leaf length (cm)	19.9	<	21.6	13.3	<	16.4	13.7	~	15.1	16.7	~	17.4	15.9	<	17.6	110.7
Maximum leaf width (cm)	12.3	<	15.0	12.9	~	13.8	14.1	~	13.4	13.4	~	15.2	13.2	<	14.4	109.1
Leaf length/width	1.62	~	1.46	1.04	~	1.20	0.98	~	1.14	1.26	~	1.19	1.22	~	1.25	102.5
Leaf area (cm^2^)	164	<	218	115	<	152	128	~	136	150	~	178	139	<	171	123.0
Leaf weight (g)	8.1	<	12.7	5.2	<	9.8	7.7	~	7.4	9.8	~	11.5	7.7	<	10.4	135.1
Pigments																
Chlorophyll (SPAD-Sqrt)	33.9	~	34.7	36.9	>	28.8	21.7	~	20.7	23.8	~	19.8	29.1	>	26.0	89.3
Anthocyanins (ACI-Lb)	36.9	~	27.8	10.9	~	11.2	7.9	~	9.6	2.5	~	2.5	16.1	~	12.8	79.5
Photosynthesis																
QY_Max	0.66	~	0.66	0.72	<	0.74	0.76	~	0.77	0.77	~	0.76	0.73	~	0.73	100.7
Fv/Fm_L1	0.66	~	0.67	0.69	<	0.71	0.74	~	0.75	0.68	<	0.70	0.69	<	0.71	102.2
NDVI (median)	0.82	~	0.83	0.72	~	0.77	0.77	~	0.74	0.75	~	0.73	0.77	~	0.77	100.3
Seed weight (g per 1,000 seeds)	0.95	<	2.07	1.21	<	1.77	1.17	<	1.32	0.89	<	1.97	1.05	<	1.78	169.5

Leaf parameters were measured on a plant’s largest leaf.

2x and 4x denote ploidy levels. Overall values combine data across tested accessions.

‘>‘ and ‘<‘ symbols indicate significant (p < 0.05) difference between two values and the direction of inequality, ‘~’ symbol indicates non-significant difference between the two values.

**Table 5:** Stomata density and size measured on adaxial leaf surfaces of greenhouse-grown plants.

Stomata trait	Annapolis			Eruption			Merlot			SM13-L2			Overall			4x/2x
	2x		4x	2x		4x	2x		4x	2x		4x	2x		4x	(%)
Young leaves																
Density (per mm^2^)	39.1	>	29.5	23.0	>	18.3	24.9	>	18.5	35.3	>	30.7	32.0	>	25.3	79.1
Length (µm)	26.0	<	35.4	29.3	<	35.8	33.8	<	52.3	37.1	~	40.9	31.7	<	41.1	129.7
Width (µm)	17.6	<	26.7	19.0	~	19.1	18.5	<	23.3	21.0	<	25.7	18.7	<	24.1	128.9
Length/width	1.49	~	1.33	1.55	~	2.05	1.85	<	2.24	1.80	~	1.59	1.70	~	1.78	104.7
Area (µm^2^)	360	<	749	437	~	533	489	<	957	607	<	830	469	<	777	165.7
Old leaves																
Density (per mm^2^)	27.1	>	10.8	17.6	>	11.0	13.6	>	11.6	27.7	>	18.0	20.8	>	12.0	57.7
Length (µm)	29.2	<	41.2	24.9	<	42.8	43.0	~	44.1	28.4	<	41.9	31.0	<	42.7	137.7
Width (µm)	17.8	<	24.1	16.1	<	25.5	19.8	<	23.0	17.9	<	24.4	17.8	<	24.3	136.5
Length/width	1.70	~	1.71	1.55	~	1.69	2.18	~	1.91	1.64	~	1.73	1.76	~	1.77	100.6
Area (µm^2^)	408	<	779	320	<	858	670	~	799	394	<	803	438	<	813	185.6

2x and 4x denote ploidy levels. Overall values combine data across tested accessions.

‘>‘ and ‘<‘ symbols indicate significant (p < 0.05) difference between two values and the direction of inequality, ‘~’ symbol indicates non-significant difference between the two values.

**Table 6:** Content of pigments, size of leaves, plants, and roots evaluated on greenhouse-grown plants at market maturity.

Trait	Annapolis			Annapolis			Eruption			Eruption			Overall			4x/2x
	Low rate			High rate			Low rate			High rate						
	2x		4x	2x		4x	2x		4x	2x		4x	2x		4x	(%)
Pigments																
Chlorophyll (SPAD-Sqrt)	35.5	>	33.1	40.3	>	38.3	30.4	>	28.6	36.0	>	34.3	35.6	>	33.6	94.5
Anthocyanins (ACI-Lb)	49.1	>	39.4	42.5	~	41.0	13.2	~	14.0	15.5	~	16.4	30.1	~	27.7	92.1
Leaf																
Number of leaves	12.6	>	9.7	13.2	>	8.9	12.9	>	11.4	12.2	>	10.5	12.7	>	10.1	79.4
Leaf length (cm)	21.2	~	22.2	22.6	~	21.8	15.9	<	19.5	17.6	<	20.8	19.3	<	21.1	109.1
Leaf width (cm)	14.3	<	17.2	16.0	<	19.6	15.6	<	17.7	15.1	<	18.3	15.3	<	18.2	119.3
Leaf thickness (µm)	203	<	219	202	<	214	259	~	271	236	~	238	225	<	236	104.7
Leaf fresh weight (g)	16.4	<	27.1	24.7	<	33.9	20.1	~	20.0	21.2	~	24.3	20.6	<	26.3	127.8
Leaf dry weight (g)	2.64	~	3.27	3.89	~	3.35	3.80	~	3.52	3.63	~	3.11	3.49	~	3.31	94.9
Plant																
Plant height (cm)	21.0	~	21.2	20.7	~	19.2	15.5	<	18.6	16.8	<	18.8	18.5	<	19.5	105.2
Plant width (cm)	29.2	~	28.4	30.3	~	30.7	25.2	<	27.4	26.0	<	30.3	27.7	<	29.2	105.5
Plant fresh weight (g)	95.5	~	94.7	141.9	~	128.0	94.4	~	93.1	133.5	~	140.3	116.1	~	114.0	98.2
Plant dry weight (g)	3.42	~	3.15	6.89	>	5.53	2.95	~	3.17	4.90	<	6.67	4.54	~	4.63	102.0
Root																
Primary root length (cm)	21.8	~	25.5	20.8	~	19.3	23.2	~	24.2	21.5	~	21.8	21.8	~	22.7	103.9
Root fresh weight (g)	22.4	>	15.0	18.0	>	13.3	14.2	~	15.9	10.0	~	12.0	16.1	>	14.0	87.1
Root dry weight (g)	4.80	>	3.87	4.77	>	3.70	4.28	~	4.34	3.98	~	3.83	4.46	>	3.93	88.3

Low fertilizer rate (40% of optimal), high fertilizer rate (160% of optimal).

2x and 4x denote ploidy levels. Overall values combine data across tested accessions.

‘>‘ and ‘<‘ symbols indicate significant (p < 0.05) difference between two values and the direction of inequality, ‘~’ symbol indicates non-significant difference between the two values.

### 3.4 Field experiments

When plants were grown in commercial fields, diploids of all accessions reached significantly more advanced developmental stage at harvest time than tetraploid lines (stage of 5.1 vs. 3.7) (**Table 7**). Diploid plants were heavier (780 g vs. 590 g), wider (27.4 cm vs. 23.2 cm), with a longer core (8.8 cm vs. 6.3 cm). Ploidy did not consistently affect plant height, as diploids of cultivar Annapolis and breeding line SM13-L2 were taller than tetraploids, white tetraploids of cultivar Eruption were taller than diploids. No significant difference was observed in the content of pigments. Tetraploid plants of all lines, however, had a substantially and significantly lower frequency of tipburn (1.8% on average) than those of diploids (22.2% on average) (**Table 7**).

**Table 7:** Content of pigments, tipburn incidence, and size of field-grown plants at market maturity.

Trait	Annapolis			Eruption			SM13-L2			Overall			4x/2x
	2x		4x	2x		4x	2x		4x	2x		4x	(%)
Plant													
Development (stage)	5.3	>	2.9	4.0	>	2.0	5.5	>	4.0	5.1	>	3.7	73.5
Height (cm)	30.4	>	28.1	20.4	<	24.8	27.7	>	24.3	26.2	~	25.8	98.5
Width (cm)	23.0	>	19.6	23.2	~	21.6	36.1	>	28.3	27.4	>	23.2	84.7
Core length (cm)	10.3	>	8.1	6.8	~	6.0	9.3	>	4.6	8.8	>	6.3	71.6
Fresh weight (g)	560	>	390	730	~	720	1,060	>	650	780	>	590	75.6
Pigments													
Chlorophyll (SPAD-Sqrt)	43.7	~	46.5	40.2	~	35.3	37.5	~	34.8	40.8	~	39.6	97.1
Anthocyanins (ACI-Lb)	425.8	~	611.0	59.3	~	57.5	4.2	~	3.7	235.2	~	302.8	129.5
Tipburn incidence (%)	6.7	>	0.0	33.3	>	5.3	26.7	>	0.0	22.2	>	1.8	8.1

2x and 4x denote ploidy levels. Overall values combine data across tested accessions.

‘>‘ and ‘<‘ symbols indicate significant (p < 0.05) difference between two values and the direction of inequality, ‘~’ symbol indicates non-significant difference between the two values.

### 3.5 Mineral elements composition

Analyses of mineral elements composition were performed on leaves and roots of greenhouse-grown plants and on leaves of field-grown plants. There was a significant difference in the overall content of 11 elements when compositions of roots from diploid and tetraploid plants were compared. Roots of diploid plants contained more Mg, Fe, Ni, Co, Al, Cd, and Ti, while roots of tetraploids had a higher content of N, P, K, and B (**Table 8**). Results from analyses of leaves of greenhouse-grown plants revealed significant differences in the content of nine compounds, all of them were higher in diploid plants (Ca, Mg, Fe, Mn, Si, Ba, Cr, Li, and Sr) (**Table 9**). Mineral element composition of leaves from field-grown plants showed an overall higher contend of Ca, Fe, Mo, Si, Al, Cd, Ag, and Ti in diploids, while tetraploids contained a higher content of Cl- (**Table 10**). Iron was consistently and significantly higher in diploids than in tetraploids at all three combinations of tissue (roots or leaves) and growing environments (greenhouse or field), while calcium and silicon were consistently higher in leaves of diploid plants regardless of the growing environment (**Tables 8**–**10**).

**Table 8:** Content of mineral elements in roots of greenhouse-grown plants at two fertilizer rates.

Element	Annapolis						Eruption						Overall			
	Low rate			High rate			Low rate			High rate						4x/2x
	2x		4x	2x		4x	2x		4x	2x		4x	2x		4x	(%)
Macronutrients (primary)																
Nitrogen	1.71	~	2.15	3.39	~	3.90	2.06	~	2.39	3.86	~	3.89	2.76	<	3.08	111.8
Phosphorus	3,482	~	4,346	7,190	<	10,315	4,378	~	4,806	7,822	<	9,681	5,718	<	7,287	127.4
Potassium	14,231	~	16,075	20,092	<	34,066	11,576	~	13,386	23,657	~	23,670	17,389	<	21,799	125.4
Macronutrients (secondary and tertiary)																
Sulfur	4,605	~	4,488	5,318	~	5,238	4,213	~	3,875	4,821	~	5,542	4,739	~	4,786	101.0
Calcium	8,087	~	8,690	8,672	~	8,029	9,010	~	9,323	8,698	~	9,006	8,617	~	8,762	101.7
Magnesium	7,390	>	6,044	6,482	>	3,798	6,163	~	5,698	3,595	~	3,669	5,907	>	4,802	81.3
Micro-nutrients																
Iron	359	~	382	585	~	456	432	~	400	920	>	556	574	>	448	78.1
Molybdenum	1.91	~	1.94	3.16	~	2.94	2.66	~	2.91	3.10	~	3.01	2.71	~	2.70	99.6
Boron	23.1	~	25.7	25.7	~	27.9	22.6	~	22.0	27.4	<	31.4	24.7	<	26.8	108.2
Copper	13.73	~	15.21	30.22	~	31.29	19.31	~	22.63	51.53	>	34.52	28.70	~	25.91	90.3
Manganese	53.5	~	59.7	118.1	~	127.9	66.2	~	60.9	283.5	>	176.7	130.3	~	106.3	81.6
Sodium	21,337	~	23,270	19,575	~	16,873	22,075	~	21,635	17,072	~	20,019	20,014	~	20,450	102.2
Zinc	95.4	~	96.4	112.3	~	106.9	91.7	~	88.2	112.9	~	106.0	103.1	~	99.4	96.5
Nickel	1.48	~	1.51	2.27	>	1.81	1.82	~	1.44	2.56	>	1.75	2.03	>	1.63	80.0
Chloride	40,607	~	36,761	17,712	~	11,870	36,191	~	31,286	10,450	~	14,669	26,240	~	23,646	90.1
Cobalt	0.45	~	0.45	1.30	~	1.18	0.62	~	0.45	2.74	>	1.74	1.28	>	0.95	74.6
Silicon	511	~	580	686	>	523	583	~	558	697	~	670	619	~	583	94.1
Vanadium	0.600	~	0.640	0.902	>	0.485	0.812	~	0.878	0.678	~	0.665	0.748	~	0.667	89.2
Selenium	1.23	~	1.23	2.83	~	3.79	1.32	~	1.38	5.81	~	5.50	2.80	~	2.98	106.5
Other elements																
Aluminum	337	~	378	484	>	371	410	~	371	568	>	467	450	>	397	88.2
Arsenic	3.16	~	3.73	4.09	>	3.16	2.93	~	2.47	3.46	~	3.35	3.41	~	3.18	93.2
Barium	11.62	~	12.93	15.72	~	14.63	12.99	~	13.18	18.13	~	17.86	14.62	~	14.65	100.2
Cadmium	0.27	~	0.25	0.38	~	0.35	0.33	~	0.28	0.49	>	0.40	0.37	>	0.32	86.3
Chromium	0.98	~	0.99	1.21	~	1.23	1.04	~	0.93	1.50	~	1.26	1.18	~	1.10	93.0
Lead	1.14	~	1.29	2.07	~	1.95	1.68	~	1.62	1.85	~	1.52	1.69	~	1.59	94.5
Lithium	5.38	~	5.55	5.33	>	4.16	5.11	~	4.57	4.08	~	4.35	4.98	~	4.66	93.6
Mercury	0.080	~	0.080	0.080	~	0.080	0.080	~	0.080	0.080	~	0.080	0.080	~	0.080	100.0
Silver	0.17	~	0.08	0.41	~	0.20	0.53	~	0.54	0.35	~	0.10	0.37	~	0.23	62.5
Strontium	63.2	~	67.7	67.8	~	66.5	67.9	~	69.0	68.8	~	73.4	66.9	~	69.1	103.3
Tin	0.70	~	0.72	0.70	~	0.70	0.70	~	0.70	0.70	~	0.75	0.70	~	0.72	102.1
Titanium	8.8	~	10.2	14.4	>	9.5	13.3	~	10.4	16.5	>	12.3	13.2	>	10.6	80.2

Low fertilizer rate (40% of optimal), high fertilizer rate (160% of optimal).

2x and 4x denote ploidy levels. Overall values combine data across tested accessions.

‘>‘ and ‘<‘ symbols indicate significant (p < 0.05) difference between two values and the direction of inequality, ‘~’ symbol indicates non-significant difference between the two values.

**Table 9:** Content of mineral elements in above ground parts of greenhouse-grown plants at two fertilizer rates.

Element	Annapolis						Eruption						Overall			
	Low rate			High rate			Low rate			High rate						4x/2x
	2x		4x	2x		4x	2x		4x	2x		4x	2x		4x	(%)
Macronutrients (primary)																
Nitrogen	2.35	~	2.63	4.31	~	4.15	2.69	~	2.65	4.42	~	4.13	3.44	~	3.39	98.6
Phosphorus	3,852	~	4,409	7,436	~	6,795	4,210	~	4,224	7,834	>	6,735	5,833	~	5,541	95.0
Potassium	36,202	<	45,121	53,386	~	57,397	44,251	~	42,572	66,230	~	63,908	50,017	~	52,250	104.5
Macronutrients (secondary and tertiary)																
Sulfur	1,947	~	1,954	2,748	~	2,825	2,020	~	1,969	3,130	~	2,846	2,461	~	2,399	97.5
Calcium	14,472	~	14,761	15,353	~	13,661	14,898	>	10,157	14,275	>	11,539	14,749	>	12,530	84.9
Magnesium	6,029	<	7,068	7,097	~	6,843	7,074	>	5,420	7,972		6,558	7,043	>	6,472	91.9
Micro-nutrients																
Iron	119	~	140	415	>	228	126	~	141	357	~	282	254	>	198	77.8
Molybdenum	0.29	~	0.25	0.48	~	0.18	0.25	~	0.22	0.20	~	0.39	0.30	~	0.26	84.8
Boron	35.3	~	38.0	38.8	<	44.7	38.7	~	39.5	46.5	~	45.9	39.8	~	42.0	105.5
Copper	7.77	~	7.52	10.04	~	10.11	7.42	~	7.46	11.53	~	9.94	9.19	~	8.76	95.3
Manganese	81.7	~	92.7	305.8	>	211.5	87.9	~	90.0	299.1	~	234.6	193.6	>	157.2	81.2
Sodium	13,851	~	13,709	9,768	~	7,659	15,027	~	16,446	7,896	~	8,212	11,636	~	11,506	98.9
Zinc	53.9	<	73.8	79.2	~	82.3	74.3	~	79.9	112.6	~	99.9	80.0	~	84.0	105.0
Nickel	0.39	~	0.31	0.42	~	0.41	0.38	~	0.34	0.49	~	0.49	0.42	~	0.39	92.3
Chloride	38,765	~	42,520	35,202	~	38,552	39,952	~	36,930	40,366	~	37,695	38,571	~	38,924	100.9
Cobalt	0.14	~	0.14	0.20	~	0.13	0.13	~	0.13	0.13	~	0.18	0.15	~	0.15	96.5
Silicon	440	~	418	419	~	358	367	>	293	360	~	316	397	>	346	87.3
Vanadium	0.060	~	0.060	0.055	~	0.056	0.060	~	0.060	0.055	~	0.055	0.058	~	0.058	100.3
Selenium	1.24	~	1.25	2.99	~	2.93	1.25	~	1.25	2.96	~	2.92	2.11	~	2.09	98.9
Other elements																
Aluminum	73	~	68	63	~	62	77	~	70	73	~	66	72	~	66	92.7
Arsenic	0.78	~	0.70	0.64	~	0.58	0.67	~	0.51	0.62	~	0.59	0.68	~	0.59	86.9
Barium	6.53	~	6.64	7.39	~	7.21	6.94	>	4.51	7.07	>	5.55	6.98	>	5.98	85.6
Cadmium	0.22	~	0.27	0.35	~	0.31	0.26	~	0.21	0.41	>	0.32	0.31	~	0.28	90.0
Chromium	0.41	~	0.37	0.41	~	0.40	0.38	~	0.35	0.39	~	0.36	0.40	>	0.37	91.9
Lead	0.80	~	0.78	0.93	~	0.91	0.78	~	0.80	0.88	~	0.89	0.85	~	0.84	99.7
Lithium	8.98	>	7.89	6.72	>	5.57	7.85	>	5.49	5.27	~	4.56	7.21	>	5.88	81.5
Mercury	0.080	~	0.080	0.075	~	0.076	0.080	~	0.080	0.075	~	0.075	0.078	~	0.078	100.2
Silver	0.05	~	0.06	0.04	~	0.04	0.08	~	0.09	0.04	~	0.04	0.05	~	0.06	106.9
Strontium	59.1	~	61.7	62.1	~	57.4	62.9	>	40.9	56.9	>	45.8	60.3	>	51.4	85.4
Tin	0.78	~	0.74	0.69	~	0.71	0.78	~	0.74	0.69	~	0.69	0.73	~	0.72	98.0
Titanium	1.7	~	1.3	1.5	~	1.5	1.4	~	1.2	1.7	~	1.5	1.6	~	1.4	87.7

Low fertilizer rate (40% of optimal), high fertilizer rate (160% of optimal).

2x and 4x denote ploidy levels. Overall values combine data across tested accessions.

‘>‘ and ‘<‘ symbols indicate significant (p < 0.05) difference between two values and the direction of inequality, ‘~’ symbol indicates non-significant difference between the two values.

**Table 10:** Content of mineral elements in above ground parts of field-grown plants.

Element	Annapolis			Eruption			SM13-L2			Overall			4x/2x
	2x		4x	2x		4x	2x		4x	2x		4x	(%)
Macronutrients (primary)													
Nitrogen	4.04	~	4.10	3.89	~	3.55	2.52	~	2.61	3.48	~	3.42	98.1
Phosphorus	5,432	~	5,700	4,724	~	4,714	4,121	~	4,567	4,759	~	4,994	104.9
Potassium	44,883	~	43,333	55,544	>	43,128	33,019	~	37,943	44,482	~	41,468	93.2
Macronutrients (secondary and tertiary)													
Sulfur	2,305	~	2,280	2,112	~	2,098	2,075	~	2,031	2,164	~	2,136	98.7
Calcium	10,575	~	9,962	9,693	>	6,531	9,119	~	7,821	9,796	>	8,105	82.7
Magnesium	4,080	~	4,167	3,778	>	2,563	3,740	~	3,069	3,866	~	3,266	84.5
Micro-nutrients													
Iron	357	~	254	402	>	262	181	~	197	313	>	238	76.0
Molybdenum	0.99	~	0.58	1.06	>	0.56	0.24	~	0.28	0.76	>	0.48	62.2
Boron	27.7	~	28.2	27.4	~	27.3	24.5	~	24.8	26.5	~	26.8	101.1
Copper	7.96	~	8.57	12.42	>	7.51	5.48	~	5.02	8.62	~	7.03	81.6
Manganese	86.7	~	88.6	101.0	~	93.0	97.4	~	76.3	95.0	~	86.0	90.4
Sodium	5,293	~	5,192	6,072	~	6,271	7,904	~	6,416	6,423	~	5,959	92.8
Zinc	30.7	~	34.7	42.9	~	39.0	28.4	~	28.5	34.0	~	34.1	100.2
Nickel	1.07	~	0.93	0.93	~	1.14	0.55	~	0.81	0.85	~	0.96	113.4
Chloride	18,070	<	29,295	16,679	~	20,729	27,671	~	29,129	20,807	<	26,384	126.8
Cobalt	0.13	~	0.13	0.13	~	0.13	0.13	~	0.13	0.13	~	0.13	100.0
Silicon	588	>	391	543	>	357	289	>	308	474	>	352	74.3
Vanadium	0.083	~	0.060	0.187	~	0.088	0.060	~	0.072	0.110	~	0.073	66.7
Selenium	1.23	~	1.23	1.23	~	1.23	1.23	~	1.23	1.23	~	1.23	100.0
Other elements													
Aluminum	328	~	208	390	>	237	160	~	173	293	>	206	70.3
Arsenic	0.53	>	0.43	0.41	~	0.39	0.33	~	0.34	0.43	~	0.39	90.8
Barium	8.17	~	7.59	6.82	~	5.76	4.57	~	4.85	6.52	~	6.07	93.1
Cadmium	0.88	~	0.71	1.17	>	0.72	0.70	~	0.51	0.92	>	0.65	70.4
Chromium	0.68	~	0.50	0.75	>	0.54	0.37	~	0.41	0.60	~	0.49	81.0
Lead	0.78	<	0.87	0.78	~	0.78	0.78	~	0.79	0.78	~	0.81	104.4
Lithium	1.29	~	0.96	1.36	~	0.82	1.01	~	1.02	1.22	~	0.93	76.6
Mercury	0.080	~	0.080	0.080	~	0.080	0.080	~	0.080	0.080	~	0.080	100.0
Silver	0.29	>	0.19	0.83	>	0.06	0.04	>	0.04	0.39	>	0.10	24.6
Strontium	57.0	~	56.1	51.5	~	36.5	45.2	~	39.8	51.2	~	44.1	86.1
Tin	0.70	~	0.70	0.70	~	0.70	0.72	~	0.96	0.71	~	0.79	111.5
Titanium	19.7	~	12.0	23.9	>	14.2	9.0	~	10.7	17.5	>	12.3	70.4

2x and 4x denote ploidy levels. Overall values combine data across tested accessions.

‘>‘ and ‘<‘ symbols indicate significant (p < 0.05) difference between two values and the direction of inequality, ‘~’ symbol indicates non-significant difference between the two values.

### 3.6 DNA methylation

WGBS revealed 498 DMR, 106 of them were PDMR with at least 50% difference in the level of methylation between neotetraploids and their diploid progenitors. Fifty of the PDMR were hypermethylated in diploid accessions while 56 were hypermethylated in tetraploid lines. PDMR were distributed across all nine chromosomes with 14 PDMR on chr. 1, 8 on chr. 2, 12 on chr. 3, 11 on chr. 4, 10 on chr. 5, 10 on chr. 6, 12 on chr. 7, 12 on chr. 8, and 15 on chr. 9 (**Supplemental Table 1**). Two PDMR were found in the genomic areas not yet assigned to any of the chromosomes. At least 18 of PDMR were detected in proximity of genes predicted to be involved in plant development, and reaction to biotic and abiotic stressors (**Supplemental Table 1**). For example, the most hypermethylated region of tetraploids (difference in methylation from diploids = 95%, adjusted *p* = 6.38E-30) located on chr. 5 contains predicted protein for glucan 1,3-alpha-glucosidase (LOC111897613). This enzyme has been described to play a function in cell division, pollen development, regulation of plasmodesmata signaling, reaction to abiotic stresses, pathogen defense, flower formation, and seed maturation (Doxey et al., 2007). Other notable genomic regions hypermethylated in neotetraploids contain predicted proteins for 5’-3’ exoribonuclease, G-type lectin S-receptor-like serine/threonine-protein kinase, BTB/POZ and TAZ domain-containing protein, phosphatidyl glycerophosphate phosphatase PTPMT2, cyclin-dependent kinases regulatory subunit, and endoplasmic reticulum oxidoreductin. On the other hand, genomic regions significantly more hypermethylated in diploid accessions contained predicted proteins for homeobox-leucine zipper protein HAT5, SNF1-related protein kinase catalytic subunit alpha KIN10, G-type lectin S-receptor-like serine/threonine-protein kinase, GDSL esterase/ipase, ubiquitin-protein ligase BOI, histone-arginine methyltransferase, and zinc finger A20 and AN1 domain-containing stress-associated protein (**Supplemental Table 1**).

## 4 Discussion

Thought the production of neotetraploids using colchicine treatment is a well-established (Winkler, 1916) and frequently used method (Eng and Ho, 2019), a successful production of tetraploids may not always be a straightforward process. Different species or even cultivars of the same species differ in their response to colchicine (Nair, 2004), therefore, optimization of the treatment may be needed. In four accessions described in the current study, the frequency of neotetraploids occurring after the colchicine treatment ranged from 6.7% in Eruption to 25.0% in SM13-L2. Other two cultivars initially tested in our laboratory using the same colchicine treatments produced neotetraploids at the rate of 1% (cultivar Salinas) and 20% (cultivar Hearts Delight). A similar range of tetraploids were obtained in other plant species; e.g., up to 24.8% in thyme (*Thymus vulgaris*) (Mohammadi et al., 2023). Another complication of using colchicine is its uneven effect on treated tissue thus leading to production of plants with a range of ploidy levels (Eng and Ho, 2019) as was also observed in our study (**Figure 1**). Moreover, some of the tetraploid plants do not produce seeds or their seeds are sterile (Einset, 1944; Einset, 1947). Additionally, induced tetraploids can spontaneously revert to their diploid level (Eng and Ho, 2019). Therefore, we have used flow cytometry to test ploidy of plants at each generation, the approach that allowed us to detect and to use for experiments only stable tetraploid lines. We also carried out regular phenotypic comparisons of tetraploid plants with their diploid progenitors at each generation to minimize a chance of performing experiments on lines with colchicine-induced mutations (Eenink and Groenwold, 1981; Datta, 2009).

Our wide-ranging analyses confirmed previously reported phenotypic differences between lettuce neotetraploids and their diploid progenitors, particularly larger leaves (Thompson, 1939), flower heads, stomata, and seeds (Eenink and Alvarez, 1975; Eenink, 1980), delayed seed development (Thompson, 1939), fewer seeds (Einset, 1944; Eenink and Alvarez, 1975; Eenink, 1980), fewer stomata per leaf area (Eenink and Alvarez, 1975; Eenink, 1980), and less marginal frilling and savoying (Thompson, 1939) in tetraploids (**Tables 3**–**6**). Also, when plants were grown in commercial fields (**Table 7**), tetraploid plants were lighter than diploid ones (Reinink and Blom-Zandstra, 1989) at harvest.

Besides confirming previously reported differences, we have also observed that tetraploid seedlings were generally heavier (**Table 1**) and had a more rapid early development (**Table 2**). It is not possible to determine from the current results alone whether observed differences were directly related to the ploidy level or resulted from an indirect effect of a seed size (neotetraploids produce only a few, but substantially heavier seeds). It has been shown that lettuce seed weight corelates positively with both seed vigor (Smith et al., 1973) and subsequent plant growth (Scaife and Jones, 1970). When a growth of diploid and synthetic tetraploid *Vicia cracc*a plants was compared two weeks after planting, plant height was significantly affected by weight of planted seeds but not by their ploidy level (Münzbergová, 2017). The effect of these two factors reversed at eight weeks after planting when the number of leaves per plant was independent of the seed size but was significantly affected by ploidy (Münzbergová, 2017).

Root biomass of tetraploid plants grown in a greenhouse was overall lower on tetraploids than on diploids, though substantial differences were observed among tested genotypes (**Table 1**). There are no previous reports on root size in tetraploid lettuce, but when the morphological and cytological characteristics were measured in tetraploid and diploid hybrid sweetgum (*Liquidambar styraciflua* × *L. formosana*) on 25 days old *in vitro* plants, roots of tetraploid plants were significantly shorter with fewer root cells of a larger size and irregular shape (Chen et al., 2022). Synthesis of auxins, gibberellin, and brassinolide and signal transduction genes involved in organ elongation were downregulated in tetraploids which may explain their slower growth (Chen et al., 2022).

Comparative analysis of pigments (chlorophyll and anthocyanins) and the performance of photosynthetic apparatus indicated a generally higher content of chlorophyll (SPAD-Sqrt) in diploid accessions compared with tetraploid lines, particularly when plants were grown in a greenhouse (**Tables 4**, **6**, and **7**), while the efficiency of photosystem II (Fv/Fm) was higher in tetraploid lines (**Table 4**). Higher Fv/Fm values were previously reported in tetraploids of several plant species, including *Acer buergerianum* (Wang et al., 2021), *Anoectochilus roxburghii* (Huang et al., 2022), and hybrid poplar clones (Zhao et al., 2015), but not in *Lillium* hybrids (Cao et al., 2018) or *Citrus wilsonii* (Jiang et al., 2022). Similarly variable results across plant species were found for the content of chlorophyll with higher levels detected in tetraploids of *Lillium* (Cao et al., 2018), *A. buergerianum* (Wang et al., 2021), *C. wilsonii* (Jiang et al., 2022), comparable levels in tetraploids and diploids of *A. roxburghii* (Huang et al., 2022), and higher levels in diploids of *Cnidium officinale* (Kim et al., 2021). Studies on *Populus* hybrids revealed that some of the differences among species and experiments may be related to the physiological age of evaluated leaves. While in younger leaves the chlorophyll content (measured as SPAD-Sqrt) was higher in tetraploids, in older leaves it was higher in diploid plants (Xu et al., 2020). This difference in chlorophyll content was caused by an accelerated chloroplast degradation in ageing tetraploid leaves (Xu et al., 2020).

Analysis of lettuce roots and leaves revealed that the level of ploidy had a significant effect on their mineral element composition (**Tables 8**–**10**). The most consistent results across plant tissue (roots or leaves) and growing environment (greenhouse or field) were detected for the content of iron that was always significantly higher in diploids than in tetraploids. Iron is required for chlorophyll synthesis (Schmidt et al., 2020) thus it is possible that its content may be related to a higher chlorophyll content in diploid lettuce. Calcium and silicon were consistently higher in leaves of diploid plants as compared with tetraploid plants regardless of the growing environment. Calcium is an essential macronutrient (Thor, 2019) while silicon is regarded as quasi-essential or non-essential for plant growth and development (Luyckx et al., 2017). Both elements, however, are vital components of cell walls and play a role in resistance to exogenous stressors (Luyckx et al., 2017; Thor, 2019). It is important to note, that calcium deficiency in developing leaves causes necrotic lesions on their margins, an economically relevant physiological disorder termed tipburn (Macias-González et al., 2019). In our field experiments, tipburn was substantially more frequently observed on diploids (overall average of 22.2%) than on tetraploid plants (overall average of 1.8%) (**Table 7**). This may be an unexpected observation considering that tetraploids contained significantly lower levels of calcium in leaf tissue than their diploid counterparts. However, translocation of calcium (Beacham et al., 2023) to leaf margins rather than an absolute content of calcium in plants is thought to be associated with tipburn. Insufficient translocation and a higher tipburn frequency usually happens in the environmental conditions that promote rapid plant growth (Cox et al., 1976). Because tetraploid plants developed slower than diploids when nearing market maturity (**Table 7**), calcium translocation to their leaf margins may have been sufficiently high to prevent frequent tipburn occurrence. In addition, chromosome doubling may have affected the expression of genes involved in ion transport (Tan et al., 2015). For example, plants of hexaploid *Ipomea trifida* had a considerably higher Ca^2+^ influx in their elongating and mature root zones than diploid plants of the same species when exposed to elevated salinity (Liu et al., 2019). We have identified PDMR on chr. 2, the genomic region hypermethylated in diploid accessions that is associated with reticulon-like protein (LOC111884021). Reticulons are membrane-spanning proteins affecting endoplasmic reticulum and being involved in modulating intracellular calcium concentration (Nziengui and Schoefs, 2009; Jozsef et al., 2014). Because of the substantial effect of ploidy on frequency of tipburn, tetraploids may be considered, together with their diploid counterparts, for studying intrinsic and extrinsic factors affecting tipburn occurrence in lettuce.

Our results document that neotetraploid lettuce plants grow more rapidly than diploids (**Table 2**) at early stages but are slower to reach market maturity (**Table 7**) and more then 35 days behind diploids in producing mature seeds (**Table 3**). It is not fully understood why these differences occur and why different plant species show a dissimilar growth pattern when tetraploids are compared to their diploid progenitors. For example, a slower vegetative growth rate of tetraploids than diploids was observed in *Populus* (Xu et al., 2020) while in *Acacia senegal* diploids grew more rapidly than tetraploids (Diallo et al., 2016). There are several factors that could contribute to differences in plant growth in tetraploids due to molecular and physiological adjustments occurring when the number of chromosomes in a genome double. Neotetraploids need to adjust to the genome-dosage effect, gene redundancy, nuclear enlargement, complex partitioning of chromosomes during cell division, and epigenic remodeling that can lead to silencing or activation of genes (Adams and Wendel, 2005; Comai, 2005; Zhang et al., 2015). It was determined that changes in methylation could play a prominent role in adapting to genome shock following chromosome doubling (Zhang et al., 2015). While some of these changes associated with epigenetic changes may be potentially beneficial, others are possibly disruptive and cause instability of the neopolyploids (Comai, 2005). The WGBS performed on lettuce neotetraploids and their diploid progenitors revealed 106 PDMR; at least 18 of them associated with genes involved in plant growth and developments (**Supplemental Table 1**). It is plausible that some of these methylation changes lead to differences in plant growth, but more tests need to be performed to provide functional evidence of this supposition.

## 5 Conclusions

**Figure 5:**
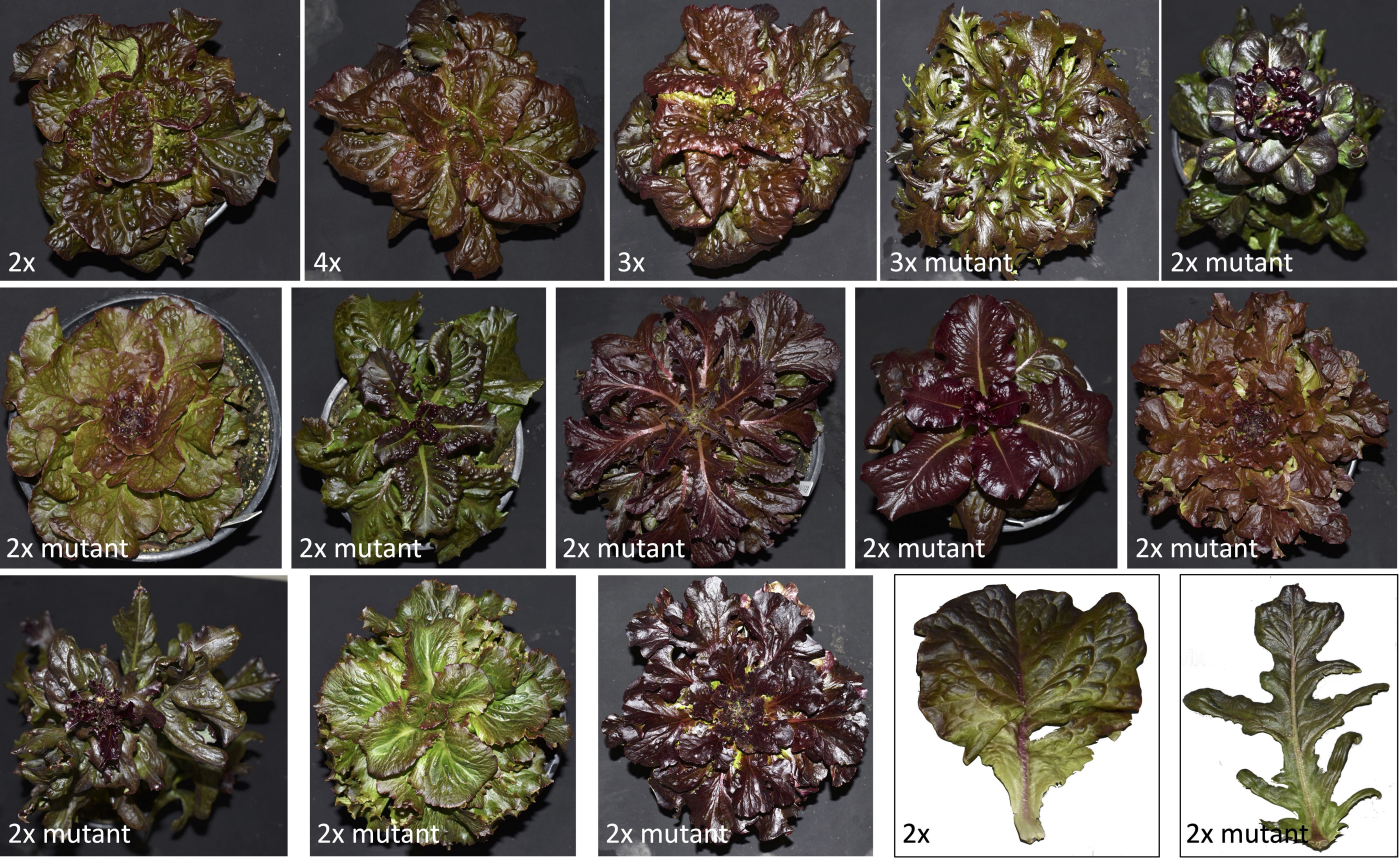
Diploid (2x), tetraploid (4x), and triploid (3x) plants of cultivar Eruption together with colchicine-induced mutants. These colchi-mutants displayed phenotypic differences in shape of leaves, pigments level, number of leaves per plant, plant architecture, and/or plant growth.

We have used colchicine treatment to successfully create somatic neotetraploids of four lettuce genotypes that showed stable ploidy and seed production for at least three generations. Compared with diploids, neoteraploids had generally a more rapid growth in early developmental stages but were slower to reach market maturity and to produce seeds. Tetraploid plants had generally a fewer but larger leaves, stomata, and seeds. Particularly the size of stomata and their density were good indicators of ploidy level. Mineral composition of plants was altered by ploidy with tetraploids containing less iron and also having a lower content of calcium and silicon in leaves. Noteworthy, despite having a lower calcium content, tetraploid plants had less frequent occurrence of tipburn than their diploid counterparts. Because of the very limited seed production, neotetraploids developed in this study are not suitable for commercial production, though their rapid growth at early stages would be valuable when harvesting young plants, such as those used in baby-leaf products, and the delayed transition to reproductive stage would be useful for extended harvest of matured heads (Simko et al., 2014b). Current tetraploid lines can be used, however, in research to study e.g., the factors contributing to tipburn, relationship between leaf internalization by *Escherichia coli* or *Salmonella enterica* and stomatal pore traits (Jacob and Melotto, 2020), and the effect of ploidy on resistance to environmental stressors (Van de Peer et al., 2021). After the treatment of seeds with colchicine we have also identified several diploid colchi-mutants with altered phenotypes (**Figure 5**), such as color, leaf shape, leaf margin, and the plant developmental rate. Such colchicine-induced lettuce mutants previously shoved a stable heritability of a mutated phenotypic trait (Eenink and Groenwold, 1981). Both colchi-mutants and neotetraploids developed during the course of this research have been incorporated into our germplasm and will be used in additional research studies and/or breeding programs.

## Data availability statement

The original contributions presented in the study are included in the article/**Supplementary Material**. Further inquiries can be directed to the corresponding author. No potentially identifiable images or data are presented in this study.

## Author contributions

IS: Conceptualization, Data curation, Formal analysis, Funding acquisition, Investigation, Methodology, Project administration, Resources, Supervision, Validation, Visualization, Writing – original draft, Writing – review & editing. RZ: Investigation, Methodology, Writing – review & editing, Formal analysis, Visualization.

## Supplementary material

Supplementary Table 1 Locations of 498 differentially methylated regions (DMR) detected using whole genome bisulfite sequencing (WGBS). The pivotal DMR (PDMR) are those with an absolute difference in percent methylation between diploids and neotetraploids larger than 50. Positive values in methylation difference indicate hypermethylation in diploids, while negative values indicate hypermethylation in neotetraploids.

## References

[B1] Adams K. L. Wendel J. F. (2005). Polyploidy and genome evolution in plants. Curr. Opin. Plant Biol. 8, 135–141. doi: 10.1016/j.pbi.2005.01.001 15752992

[B2] Adhikari N. D. Simko I. Mou B. (2019). Phenomic and physiological analysis of salinity effects on lettuce. Sensors 19, 4814. doi: 10.3390/s19214814 31694293 PMC6864466

[B3] Akalin A. Kormaksson M. Li S. Garrett-Bakelman F. E. Figueroa M. E. Melnick A.. (2012). methylKit: a comprehensive R package for the analysis of genome-wide DNA methylation profiles. Genome Biol. 13, R87. doi: 10.1186/gb-2012-13-10-r87 23034086 PMC3491415

[B4] Beacham A. M. Wilkins K. A. Davies J. M. Monaghan J. M. (2023). Vacuolar Ca^2+^/H^+^ exchanger and Ca^2+^ -ATPase homologues are differentially regulated in tipburn-resistant and susceptible lettuce (*Lactuca sativa*) cultivars. Plant Physiol. Biochem. 201, 107792. doi: 10.1016/j.plaphy.2023.107792 37285692

[B5] Cao Q. Zhang X. Gao X. Wang L. Jia G. (2018). Effects of ploidy level on the cellular, photochemical and photosynthetic characteristics in *Lilium* FO hybrids. Plant Physiol. Biochem. 133, 50–56. doi: 10.1016/j.plaphy.2018.10.027 30390431

[B6] Chen S. Zhang Y. Zhang T. Zhan D. Pang Z. Zhao J.. (2022). Comparative transcriptomic, anatomical and phytohormone analyses provide new insights into hormone-mediated tetraploid dwarfing in hybrid sweetgum (*Liquidambar styraciflua* × *L. formosana*). Front. Plant Sci. 13, 924044. doi: 10.3389/fpls.2022.924044 35832220 PMC9271929

[B7] Comai L. (2005). The advantages and disadvantages of being polyploid. Nat. Rev. Genet. 6, 836–846. doi: 10.1038/nrg1711 16304599

[B8] Cox E. F. McKee J. M. T. Dearman A. S. (1976). The effect of growth rate on tipburn occurence in lettuce. J. Hortic. Sci. 51, 297–309. doi: 10.1080/00221589.1976.11514693

[B9] Datta S. K. (2009). “A report on 36 years of practical work on crop improvement through induced mutagenesis,” in Induced plant mutations in the genomic era. Ed. Shu Q. Y. (Rome, Italy: Food and Agriculture Organization of the United Nations (FAO).

[B10] Dermen H. Emsweller S. L. (1961). The use of colchicine in plant breeding. Crops Research U.S. Department Agriculture Agric. Res. Service ARS 34 - 24, 1–10.

[B11] Diallo A. M. Nielsen L. R. Kjær E. D. Petersen K. K. Ræbild A. (2016). Polyploidy can confer superiority to West African *Acacia Senegal* (L.) Willd. trees. Front. Plant Sci. 7, 821. doi: 10.3389/fpls.2016.00821 27379120 PMC4906048

[B12] Doxey A. C. Yaish M. W. F. Moffatt B. A. Griffith M. McConkey B. J. (2007). Functional divergence in the *Arabidopsis* β-1, 3-glucanase gene family inferred by phylogenetic reconstruction of expression states. Mol. Biol. Evol. 24, 1045–1055. doi: 10.1093/molbev/msm024 17272678

[B13] Eenink A. H. (1980). Plant characteristics for distinction of diploid, triploid and tetraploid lettuce. Scientia Hortic. 12, 109–115. doi: 10.1016/0304-4238(80)90117-X

[B14] Eenink A. H. Alvarez J. M. (1975). Indirect selection for tetraploidy in lettuce (*Lactuca sativa* L.). Euphytica 24, 661–668. doi: 10.1007/BF00132904

[B15] Eenink A. H. Groenwold R. (1981). Increase of genetic variation in some lettuce cultivars by induction of mutations: A technical note. Euphytica 30, 735–737. doi: 10.1007/BF00038802

[B16] Einset J. (1944). Cytological basis for sterility in induced autotetraploid lettuce (*Lactuca sativa* L.). Am. J. Bot. 31, 336–342. doi: 10.1002/j.1537-2197.1944.tb08039.x

[B17] Einset J. (1947). Aneuploidy in relation to partial sterility in autotetraploid lettuce (*Lactuca sativa* L.). Am. J. Bot. 34, 99–105. doi: 10.1002/j.1537-2197.1947.tb12964.x

[B18] Eng W.-H. Ho W.-S. (2019). Polyploidization using colchicine in horticultural plants: A review. Scientia Hortic. 246, 604–617. doi: 10.1016/j.scienta.2018.11.010

[B19] Engler D. E. Grogan R. G. (1984). Variation in lettuce plants regenerated from protoplasts. J. Heredity 75, 426–430. doi: 10.1093/oxfordjournals.jhered.a109978

[B20] Forrest H. (1962). Instructive micro-replicas from nail polish. Am. Biol. Teacher 24, 523–525. doi: 10.2307/4440080

[B21] Hiraoka T. (1967). Production of polyploidy lettuce by colchicine treatment. Kanagawa Hortic. Experiment Station Bull. 15, 59–64.

[B22] Huang X. Ouyang K. Luo Y. Xie G. Yang Y. Zhang J. (2022). A comparative study of characteristics in diploid and tetraploid *Anoectochilus roxburghii* . Front. Nutr. 9, 1034751. doi: 10.3389/fnut.2022.1034751 36419553 PMC9676492

[B23] Inderbitzin P. Christopoulou M. Lavelle D. Reyes-Chin-Wo S. Michelmore R. W. Subbarao K. V.. (2019). The *LsVe1L* allele provides a molecular marker for resistance to *Verticillium dahliae* race 1 in lettuce. BMC Plant Biol. 19, 305. doi: 10.1186/s12870-019-1905-9 31291883 PMC6621938

[B24] Jacob C. Melotto M. (2020). Human pathogen colonization of lettuce dependent upon plant genotype and defense response activation. Front. Plant Sci. 10, 1769. doi: 10.3389/fpls.2019.01769 32082340 PMC7002439

[B25] Jaranowski J. Kalasa M. (1971). Comparative analysis of fertility in several *Trifolium, Melilotus, Medicago* and *Trigonella* species and forms on a di-and tetraploid level. Genetica Polonica 12, 1–16.

[B26] Jiang J. Yang N. Li L. Qin G. Ren K. Wang H.. (2022). Tetraploidy in *Citrus wilsonii* enhances drought tolerance via synergistic regulation of photosynthesis, phosphorylation, and hormonal changes. Front. Plant Sci. 13, 875011. doi: 10.3389/fpls.2022.875011 35574073 PMC9096895

[B27] Jozsef L. Tashiro K. Kuo A. Park E. J. Skoura A. Albinsson S.. (2014). Reticulon 4 is necessary for endoplasmic reticulum tubulation, STIM1-Orai1 coupling, and store-operated calcium entry. J. Biol. Chem. 289, 9380–9395. doi: 10.1074/jbc.M114.548602 24558039 PMC3969502

[B28] Kim H.-E. Han J.-E. Lee H. Kim J.-H. Kim H.-H. Lee K.-Y.. (2021). Tetraploidization increases the contents of functional metabolites in *Cnidium officinale* . Agronomy 11, 1561. doi: 10.3390/agronomy11081561

[B29] Krueger F. Andrews S. R. (2011). Bismark: a flexible aligner and methylation caller for Bisulfite-Seq applications. Bioinformatics 27, 1571–1572. doi: 10.1093/bioinformatics/btr167 21493656 PMC3102221

[B30] Lelivelt C. L. C. McCabe M. S. Newell C. A. desnoo C. B. Van Dun K. M. P. Birch-Machin I.. (2005). Stable plastid transformation in lettuce (*Lactuca sativa* L.). Plant Mol. Biol. 58, 763–774. doi: 10.1007/s11103-005-7704-8 16240172

[B31] Liu Y. Yu Y. Sun J. Cao Q. Tang Z. Liu M.. (2019). Root-zone-specific sensitivity of K^+^ -and Ca^2+^ -permeable channels to H_2_O_2_ determines ion homeostasis in salinized diploid and hexaploid *Ipomoea trifida* . J. Exp. Bot. 70, 1389–1405. doi: 10.1093/jxb/ery461 30689932 PMC6382330

[B32] Luyckx M. Hausman J.-F. Lutts S. Guerriero G. (2017). Silicon and plants: current knowledge and technological perspectives. Front. Plant Sci. 8, 411. doi: 10.3389/fpls.2017.00411 28386269 PMC5362598

[B33] Macias-González M. Truco M. J. Bertier L. D. Jenni S. Simko I. Hayes R. J.. (2019). Genetic architecture of tipburn resistance in lettuce. Theor. Appl. Genet. 132, 2209–2222. doi: 10.1007/s00122-019-03349-6 31055612

[B34] Manzoor A. Ahmad T. Bashir M. A. Hafiz I. A. Silvestri C. (2019). Studies on colchicine induced chromosome doubling for enhancement of quality traits in ornamental plants. Plants 8, 194. doi: 10.3390/plants8070194 31261798 PMC6681243

[B35] Mohammadi V. Talebi S. Ahmadnasab M. Mollahassanzadeh H. (2023). The effect of induced polyploidy on phytochemistry, cellular organelles and the expression of genes involved in thymol and carvacrol biosynthetic pathway in thyme (*Thymus vulgaris*). Front. Plant Sci. 14, 1228844. doi: 10.3389/fpls.2023.1228844 37780500 PMC10540446

[B36] Münzbergová Z. (2017). Colchicine application significantly affects plant performance in the second generation of synthetic polyploids and its effects vary between populations. Ann. Bot. 120, 329–339. doi: 10.1093/aob/mcx070 28633349 PMC5737759

[B37] Myneni R. B. Hall F. G. Sellers P. J. Marshak A. L. (1995). The interpretation of spectral vegetation indexes. IEEE Trans. Geosci. Remote Sens. 33, 481–486. doi: 10.1109/TGRS.1995.8746029

[B38] Nair R. M. (2004). Developing tetraploid perennial ryegrass (*Lolium perenne* L.) populations. New Z. J. Agric. Res. 47, 45–49. doi: 10.1080/00288233.2004.9513569

[B39] Nziengui H. Schoefs B. (2009). Functions of reticulons in plants: what we can learn from animals and yeasts. Cell. Mol. Life Sci. 66, 584–595. doi: 10.1007/s00018-008-8373-y 18989623 PMC11131481

[B40] O'Leary N. A. Wright M. W. Brister J. R. Ciufo S. Haddad D. McVeigh R.. (2016). Reference sequence (RefSeq) database at NCBI: current status, taxonomic expansion, and functional annotation. Nucleic Acids Res. 44, D733–D745. doi: 10.1093/nar/gkv1189 26553804 PMC4702849

[B41] Paysan-Lafosse T. Blum M. Chuguransky S. Grego T. Pinto B. L. Salazar G. A.. (2023). InterPro in 2022. Nucleic Acids Res. 51, D418–D427. doi: 10.1093/nar/gkac993 36350672 PMC9825450

[B42] Ramsey J. Schemske D. W. (2002). Neopolyploidy in flowering plants. Annu. Rev. Ecol. Systematics 33, 589–639. doi: 10.1146/annurev.ecolsys.33.010802.150437

[B43] Reinink K. Blom-Zandstra M. (1989). The relationship between cell size, ploidy level and nitrate concentration in lettuce. Physiologia Plantarum 76, 575–580. doi: 10.1111/j.1399-3054.1989.tb05481.x

[B44] Renny-Byfield S. Wendel J. F. (2014). Doubling down on genomes: polyploidy and crop plants. Am. J. Bot. 101, 1711–1725. doi: 10.3732/ajb.1400119 25090999

[B45] Reyes-Chin-Wo S. Wang Z. Yang X. Kozik A. Arikit S. Song C.. (2017). Genome assembly with *in vitro* proximity ligation data and whole-genome triplication in lettuce. Nat. Commun. 8, 14953. doi: 10.1038/ncomms14953 28401891 PMC5394340

[B46] Rosental L. Still D. W. You Y. Hayes R. J. Simko I. (2021). Mapping and identification of genetic loci affecting earliness of bolting and flowering in lettuce. Theor. Appl. Genet. 134, 3319–3337. doi: 10.1007/s00122-021-03898-9 34196730

[B47] Rueden C. T. Schindelin J. Hiner M. C. DeZonia B. E. Walter A. E. Arena E. T.. (2017). ImageJ2: ImageJ for the next generation of scientific image data. BMC Bioinf. 18, 529. doi: 10.1186/s12859-017-1934-z PMC5708080 29187165

[B48] Sattler M. C. Carvalho C. R. Clarindo W. R. (2016). The polyploidy and its key role in plant breeding. Planta 243, 281–296. doi: 10.1007/s00425-015-2450-x 26715561

[B49] Scaife M. A. Jones D. (1970). Effect of seed weight on lettuce growth. J. Hortic. Sci. 45, 299–302. doi: 10.1080/00221589.1970.11514359

[B50] Schmidt W. Thomine S. Buckhout T. J. (2020). Iron nutrition and interactions in plants. Front. Plant Sci. 10, 1670. doi: 10.3389/fpls.2019.01670 31998349 PMC6968163

[B51] Simko I. (2020). Predictive modeling of a leaf conceptual midpoint quasi-color (CMQ) using an artificial neural network. Sensors 20, 3938. doi: 10.3390/s20143938 32679776 PMC7412459

[B52] Simko I. (2023). Differentially methylated genomic regions of lettuce seeds relate to phenotypic divergence across horticultural types. AoB Plants 15, 1–9. doi: 10.1093/aobpla/plad060 PMC10482144 37680204

[B53] Simko I. Hasegawa D. K. Peng H. Zhao R. (2023a). Genetic and physiological determinants of lettuce partial resistance to Impatiens necrotic spot virus. Front. Plant Sci. 14, 1163683. doi: 10.3389/fpls.2023.1163683 37360711 PMC10285314

[B54] Simko I. Hayes R. J. Bull C. T. Mou B. Luo Y. Trent M. A.. (2014a). Characterization and performance of 16 new inbred lines of lettuce. HortScience 49, 679–687. doi: 10.21273/HORTSCI.49.5.679

[B55] Simko I. Hayes R. J. Mou B. McCreight J. D. (2014b). “Lettuce and spinach,” in Yield gains in major U.S. Field crops. Eds. Smith S. Diers B. Specht J. Carver B. (Madison, Wisconsin, USA: American Society of Agronomy, Inc., Crop Science Society of America, Inc., and Soil Science Society of America, Inc). doi: 10.2135/cssaspecpub33.c4

[B56] Simko I. Peng H. Sthapit Kandel J. Zhao R. (2022). Genome-wide association mapping reveals genomic regions frequently associated with lettuce field resistance to downy mildew. Theor. Appl. Genet. 135, 2009–2024. doi: 10.1007/s00122-022-04090-3 35419653

[B57] Simko I. Sthapit Kandel J. Peng H. Zhao R. Subbarao K. V. (2023b). Genetic determinants of lettuce resistance to drop caused by *Sclerotinia minor* identified through genome-wide association mapping frequently co-locate with loci regulating anthocyanin content. Theor. Appl. Genet. 136, 180. doi: 10.1007/s00122-023-04421-y 37548768

[B58] Smith O. E. Welch N. C. Little T. M. (1973). Studies on lettuce seed quality: I. Effect of seed size and weight on vigor. J. Am. Soc. Hortic. Sci. 98, 529–533. doi: 10.21273/JASHS.98.6.529

[B59] Soltis P. S. Marchant D. B. Van de Peer Y. Soltis D. E. (2015). Polyploidy and genome evolution in plants. Curr. Opin. Genet. Dev. 35, 119–125. doi: 10.1016/j.gde.2015.11.003 26656231

[B60] Sun Q. Zybailov B. Majeran W. Friso G. Olinares P. D. B. van Wijk K. J. (2009). PPDB, the plant proteomics database at Cornell. Nucleic Acids Res. 37, D969–D974. doi: 10.1093/nar/gkn654 18832363 PMC2686560

[B61] Tan F.-Q. Tu H. Liang W.-J. Long J.-M. Wu X.-M. Zhang H.-Y.. (2015). Comparative metabolic and transcriptional analysis of a doubled diploid and its diploid citrus rootstock (*C. junos* cv. Ziyang xiangcheng) suggests its potential value for stress resistance improvement. BMC Plant Biol. 15, 89. doi: 10.1186/s12870-015-0450-4 25848687 PMC4374211

[B62] The_UniProt_Consortium (2023). UniProt: the universal protein knowledgebase in 2023. Nucleic Acids Res. 51, D523–D531. doi: 10.1093/nar/gkac1052 36408920 PMC9825514

[B63] Thompson R. C. (1939). Polyploidy in lettuce induced by colchicine. Proc. Am. Soc. Hortic. Sci. 36, 641–644.

[B64] Thor K. (2019). Calcium - nutrient and messenger. Front. Plant Sci. 10, 440. doi: 10.3389/fpls.2019.00440 31073302 PMC6495005

[B65] Tong W. Li R. Huang J. Zhao H. Ge R. Wu Q.. (2021). Divergent DNA methylation contributes to duplicated gene evolution and chilling response in tea plants. Plant J. 106, 1312–1327. doi: 10.1111/tpj.15237 33730390

[B66] Van de Peer Y. Ashman T.-L. Soltis P. S. Soltis D. E. (2021). Polyploidy: an evolutionary and ecological force in stressful times. Plant Cell 33, 11–26. doi: 10.1093/plcell/koaa015 33751096 PMC8136868

[B67] Wang Y. Jia B. Ren H. Feng Z. (2021). Ploidy level enhances the photosynthetic capacity of a tetraploid variety of *Acer buergerianum* Miq. Peerj 9, e12620. doi: 10.7717/peerj.12620 35003928 PMC8684723

[B68] Wang H.-Q. Tuominen L. K. Tsai C.-J. (2011). SLIM: a sliding linear model for estimating the proportion of true null hypotheses in datasets with dependence structures. Bioinformatics 27, 225–231. doi: 10.1093/bioinformatics/btq650 21098430

[B69] Winkler H. (1916). Über die experimentelle Erzeugung von Pflanzen mit abweichenden Chromosomenzahlen. Z. für Botanik 8, 417–531.

[B70] Xu C. Zhang Y. Han Q. Kang X. (2020). Molecular mechanism of slow vegetative growth in *Populus* tetraploid. Genes 11, 1417. doi: 10.3390/genes11121417 33261043 PMC7761321

[B71] Zhang J. Liu Y. Xia E.-H. Yao Q.-Y. Liu X.-D. Gao L.-Z. (2015). Autotetraploid rice methylome analysis reveals methylation variation of transposable elements and their effects on gene expression. Proc. Natl. Acad. Sci. 112, E7022–E7029. doi: 10.1073/pnas.1515170112 26621743 PMC4687577

[B72] Zhao X. Li Y. Zheng M. Bian X. Liu M. Sun Y.. (2015). Comparative analysis of growth and photosynthetic characteristics of (*Populus simonii* × *P. nigra*) × (*P. nigra* × *P. simonii*) hybrid clones of different ploidides. PloS One 10, e0119259. doi: 10.1371/journal.pone.0119259 25867100 PMC4395098

